# A Large Fraction of Extragenic RNA Pol II Transcription Sites Overlap Enhancers

**DOI:** 10.1371/journal.pbio.1000384

**Published:** 2010-05-11

**Authors:** Francesca De Santa, Iros Barozzi, Flore Mietton, Serena Ghisletti, Sara Polletti, Betsabeh Khoramian Tusi, Heiko Muller, Jiannis Ragoussis, Chia-Lin Wei, Gioacchino Natoli

**Affiliations:** 1 Department of Experimental Oncology, European Institute of Oncology (IEO) Campus IFOM-IEO, Milan, Italy; 2 Genomics Laboratory, Wellcome Trust Centre for Human Genetics (WTCHG), University of Oxford, Oxford, United Kingdom; 3 Genome Technology and Biology Group, Genome Institute of Singapore, Singapore; University of Queensland, Australia

## Abstract

Mammalian genomes are pervasively transcribed outside mapped protein-coding genes. One class of extragenic transcription products is represented by long non-coding RNAs (lncRNAs), some of which result from Pol_II transcription of bona-fide RNA genes. Whether all lncRNAs described insofar are products of RNA genes, however, is still unclear. Here we have characterized transcription sites located outside protein-coding genes in a highly regulated response, macrophage activation by endotoxin. Using chromatin signatures, we could unambiguously classify extragenic Pol_II binding sites as belonging to either canonical RNA genes or transcribed enhancers. Unexpectedly, 70% of extragenic Pol_II peaks were associated with genomic regions with a canonical chromatin signature of enhancers. Enhancer-associated extragenic transcription was frequently adjacent to inducible inflammatory genes, was regulated in response to endotoxin stimulation, and generated very low abundance transcripts. Moreover, transcribed enhancers were under purifying selection and contained binding sites for inflammatory transcription factors, thus suggesting their functionality. These data demonstrate that a large fraction of extragenic Pol_II transcription sites can be ascribed to *cis*-regulatory genomic regions. Discrimination between lncRNAs generated by canonical RNA genes and products of transcribed enhancers will provide a framework for experimental approaches to lncRNAs and help complete the annotation of mammalian genomes.

## Abstract 2

A substantial fraction of extragenic Pol II transcription sites coincides with transcriptional enhancers, which may be relevant for functional annotation of mammalian genomes.

## Author Summary

Mammalian genomes contain vast intergenic regions that are extensively transcribed and generate various types of short and long non-coding RNAs (ncRNAs). Although in some cases specific functions have been assigned to intergenic transcripts, the functional significance of this transcriptional output remains largely unknown, and the possibility exists that part of this transcription reflects noise generated by random collisions of the transcriptional machinery with the genome to generate meaningless transcription. In this study we used chromatin signatures to characterize extragenic transcription sites targeted by RNA Polymerase II (RNA Pol II) in a highly regulated response—endotoxin activation of macrophages. We found that a significant portion of extragenic transcription sites are associated with the chromatin signature characteristic of enhancers. Consistent with their chromatin signature, we found that these extragenic transcription sites are under purifying selection and contain binding sites for inflammatory transcription factors, as well as for PU.1, a hematopoietic transcription factor that marks enhancers in macrophages. Moreover, much of this extragenic transcription is regulated by stimulation. We also identified hundreds of transcribed regions with a signature of canonical RNA genes. Our data indicate that extragenic transcription sites can be efficiently classified using chromatin signatures, which will be relevant for functional annotation of mammalian genomes.

## Introduction

A most striking finding of modern genomic biology has been the identification of a large amount of transcription that occurs outside mapped protein-coding genes and generates a heterogeneous spectrum of transcripts [1],[2], which may in principle exert broad regulatory or effector functions [3]–[5]. These data imply that the amount of information contained in the complex genomes of eukaryotes, and higher eukaryotes in particular, is much higher than the classical linear models of genomic organization can accommodate [6]. The abundance of non-coding transcription also generates novel conceptual and experimental challenges. Probably the most outstanding and urgent issues are (i) to define how many, and which, of the transcriptional events occurring outside protein-coding genes are functional and regulated (as opposed to those that represent noise) [7],[8]; (ii) to discriminate if functionality is conveyed by the transcript, by the act of transcription, or both; (iii) to classify functional transcription sites as canonical RNA genes or regulatory sequences undergoing transcription, like enhancers and locus control regions (LCRs), that in anecdotal cases were shown to be transcribed and to generate ncRNAs [9]–[13].

Regarding functionality, the two extreme views are that most of this extragenic non-coding transcription merely represents noise, namely the consequence of unscheduled but productive collisions of RNA polymerases with random genomic regions, and that most of the products of non-coding transcription are functional RNA molecules exerting downstream functions [3],[7]. Examples of transcriptional noise may be represented both by the recently described “ripples” of transcription extending from one protein coding-gene into the adjacent genomic regions [14] and by the spurious intragenic transcription initiation events, which in yeast seem to be actively suppressed [15]. In several cases, including the Xist, Air, and Kcnq1ot1 ncRNAs [12],[16]–[23], specific functions have been ascribed to selected lncRNAs on the basis of loss- or gain-of-function experiments. Evidence for functionality of lncRNAs as a class also stems from evolutionary analyses indicating that purifying selection has acted on both the promoters and the internal sequences of lncRNA genes to eliminate nucleotide substitutions, insertions and deletions [3],[8],[24],[25]. Two aspects of these evolutionary signatures of functionality deserve a more detailed analysis. First, the overall level of conservation, albeit significant, is comparatively low, with point mutations occurring with a frequency about 10-fold higher in lncRNA sequences as compared to protein-coding genes, although lncRNA splice sites tend to be conserved [24],[25]. Second, conservation was found to be much higher at promoters than within the transcript sequences [24],[26], which may indicate either the stronger sequence constraints of regulatory regions as compared to the ncRNA products or that at least in some cases the target of purifying selection may be represented by the act of transcription rather than by its products.

The concept that transcription has roles other than generating functional products mainly stems from the analysis of *cis*-regulatory elements like LCRs and enhancers. Unidirectional transcription of the β-globin LCR by RNA Pol_II [9] is required to generate and maintain an open chromatin domain [10]. Similarly, the switch of polycomb group response elements (PRE) from a repressed to an activated state in *Drosophila* requires intergenic transcription through the PRE, indicating that in some cases transcription may provide an anti-silencing mechanism [27]. Additional examples of non-coding transcription correlating with (and causing) locus activation were described in the LCR of the major histocompatibility complex II locus [28], in the T cell receptor locus [11], and upstream of the lysozyme gene in activated macrophages [13]. Non-coding transcription occurring close to protein-coding genes also has the potential to cause gene repression. Transcription of the non-coding gene *SRG1* through the promoter of *SER3* in yeast interferes with binding of transcription factors and subsequent activation, thus providing a paradigmatic example of transcriptional interference mediated by non-coding transcription [29]. Similarly, the *Ubx* gene in *Drosophila* is repressed by non-coding transcription elongating from the upstream *bxd* locus, which results in complementary and non-overlapping patterns of expression of *Ubx* mRNA and *bxd* ncRNAs [30]. In some (but not all) cases described above, formal evidence was provided that the act of transcription *per se* (rather than the transcripts) mediates downstream effects. For instance, intergenic transcription extending in the yeast *PHO5* promoter is required for nucleosome eviction and gene activation; however, increasing the level of the unstable lncRNA generated in this region didn't affect gene activation [31]. In other cases the lncRNA generated by extragenic transcription was found to impart regulation. For instance, nascent ncRNAs were shown to act as platforms for the recruitment of an RNA-binding transcriptional regulator upstream of the *CCND1* gene [20], and the Evf2 ncRNA (derived from an ultraconserved regulatory region) was shown to act in *trans* to coactivate the homeodomain TF Dlx-2 [12].

Mechanistically, transcriptional elongation causes a broad spectrum of effects to the underlying chromatin template, including chromatin remodeling, nucleosome eviction, and changes in the acetylation and methylation state of histone tails [32],[33], effects that are all due to the association of multiple enzymatic activities with the elongating Pol_II complex [34],[35]. Direct biochemical and genetic evidence supporting this type of mechanism comes from a recent time-resolved analysis in *S. Pombe*: transiently inducible non-coding Pol_II transcription upstream of the *fbp1* locus caused a wave of chromatin remodeling preceding, and required for, binding of activating transcription factors to cognate sites in the *fbp1* promoter [36]. However, the possible role of the nascent, very low abundance ncRNAs generated by transcription upstream of *fbp1* was not directly addressed.

In spite of all these observations, it is still unclear to what extent each of these reports represents an anecdotal description of uncommon gene regulatory mechanisms or conversely a paradigmatic example of a more general contribution of non-coding transcription to gene control. Moreover, the extent to which transcription occurring outside protein-coding genes indicates underlying RNA genes rather than Pol_II elongation along distant *cis*-regulatory regions (like enhancers and LCRs) is completely unknown.

Here we took advantage of a dataset of extragenic Pol_II sites in a model of highly regulated gene expression (endotoxin-stimulated primary macrophages). Using chromatin signatures we discriminated between transcribed enhancers and transcription start sites (TSS) of RNA genes. Remarkably, 70% of extragenic transcription sites (which were frequently up- or down-regulated by endotoxin stimulation) corresponded to genomic regions with an enhancer-type chromatin signature. These Pol_II peaks overlapped with annotated lncRNAs, were associated with binding sites for inflammatory transcription factors, and displayed enhancer activity in reporter assays. We also identified about 700 extragenic Pol_II clusters with a typical signature of active TSS and highly enriched for CpG islands, thus likely representing the 5′ end of bona fide RNA-coding genes. Overall, enhancers overlap a sizeable fraction of extragenic transcription sites in higher eukaryotes.

## Results

### Regulated Extragenic Transcription Upstream of LPS-Inducible Genes

We first determined the genomic distribution of RNA Pol_II in unstimulated and activated mouse macrophages (stimulated for 2 h with LPS in the presence of gamma interferon, γIFN). These ChIP-Sequencing datasets (described in [37]) were generated with an antibody recognizing all isoforms of the large RNA Pol_II subunit, Rbp1, irrespective of their phosphorylation state. Therefore, they provide a snapshot of global Pol_II distribution over the mouse genome.

**Figure 1:**
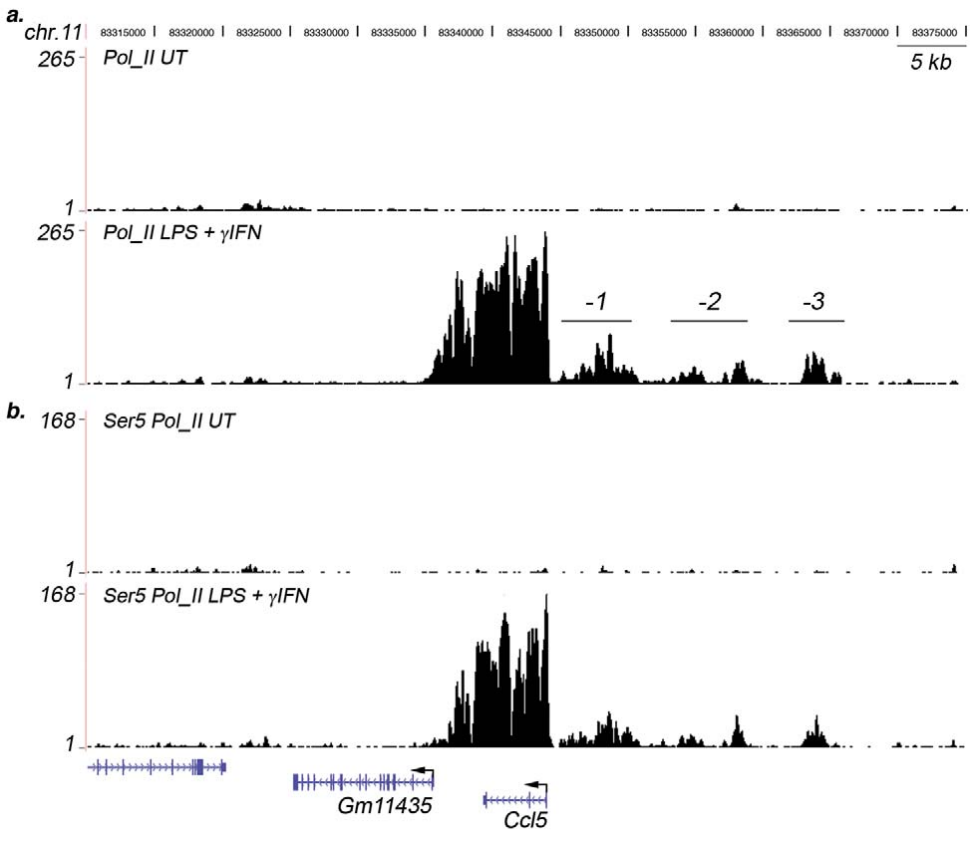
Sites of regulated extragenic transcription upstream of LPS-inducible genes. (A) Pol_II ChIP-Seq data from unstimulated and LPS+γIFN-stimulated macrophages at the Ccl5 gene and surrounding genomic regions. The extragenic Pol_II peaks (indicated as *−1*, *−2*, and *−3*) and the genomic annotations (mm9) are shown. The *y*-axis indicates the number of ChIP-Seq tags. (B) Phosphorylated Ser5 Pol_II ChIP-Seq data at the same genomic region. UT, untreated macrophages.

We first browsed genomic regions containing genes regulated by LPS stimulation, like cytokine and chemokine genes, to identify sites of extragenic transcription. Figure 1A shows an example of extragenic Pol_II sites induced by LPS stimulation and located upstream of the inflammatory chemokine gene Ccl5. Upstream Pol_II peaks are extremely broad, covering about 20 kb of extragenic sequence with no annotation of known or predicted exons; moreover their height is much lower than that found inside the coding region. Upstream Pol_II signals do not seem to be continuous (with three or four distinct clusters) and stop just upstream of the Ccl5 TSS. Upstream of another chemokine gene, Cxcl11 (Figure S1), two discrete inducible peaks can be observed, covering an area of about 10 kb. Although these peaks overlap a gene (Art3) that extends in antisense orientation over Cxcl11 (and the closely spaced Cxcl10), they cannot be ascribed to the activity of Art3, which is very poor in these cells (as indicated by the very small amount of Pol_II loaded on its TSS). Intergenic Pol_II (with no continuity with the Pol_II signals tracking from the 3′ of Cxcl11) can also be detected in the space separating the 3′ of Cxcl11 from the 5′ of Cxcl10. Other examples are shown in Figure S1.

To determine if Pol_II is actively transcribing these extragenic regions, we also generated ChIP-Seq datasets using an antibody specific for an elongating Pol_II isoform (phosphorylated at Ser5 of the carboxy-terminal domain of Rbp1) [38]. Ser5 profiles confirmed that Pol_II binding upstream of Ccl5 reflects active transcription (Figure 1B).

### Upstream Extragenic Transcription Frequently Precedes the Induction of the Adjacent Coding Gene

**Figure 2:**
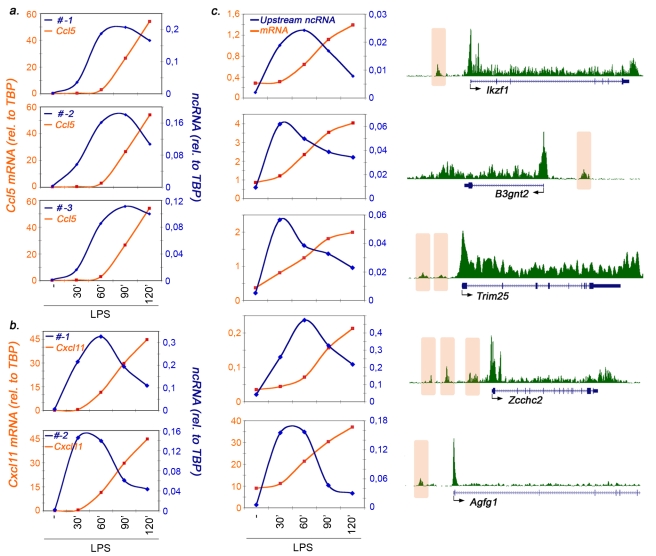
Inducible upstream extragenic transcription frequently precedes the activation of the adjacent protein-coding gene. Kinetics of induction of Ccl5 (A) and Cxcl11 (B) mRNAs relative to those of the upstream extragenic transcripts. Extragenic Ccl5 transcripts (#*−1*, *−2*, and *−3*) correspond to the Pol_II peaks shown in Figure 1. Cxcl11 transcripts #−1 and #−2 correspond to the regions in Figure S1A. Cells were stimulated with LPS+γIFN as indicated. *y*-axes indicate mRNA (left) or ncRNA (right) levels relative to those of a housekeeping gene (TBP). (C) Kinetics of mRNA induction of a panel of protein-coding genes together with the associated extragenic transcripts. The corresponding Pol_II ChIP_seq data (2h LPS+γIFN stimulation) are shown on the right. Shaded areas indicate the extragenic Pol_II peaks. For Trim25 and Zcchc2, amplicons correspond to the Pol_II peak closest to the 5′ of the gene.

To start characterizing the properties of the extragenic transcription described above, we first analyzed kinetics of induction of the corresponding ncRNA relative to that of the downstream coding gene. We carried out quantitative RT-PCR with primers designed in regions contained within the extragenic Pol_II peaks. In the case of Ccl5 we explored the three regions of extragenic transcription (named −*1*, −*2*, and −*3*) indicated in Figure 1A. Importantly, the Q-PCR primers were designed at a fixed distance in order to generate products of 200 nucleotides. Therefore, a positive signal implies the existence of RNA species of at least 200 nt. The kinetics of activation of these regions, as evaluated by the behavior of the corresponding transcripts (Figure 2A), were very similar with each other, appearing already at 30′ after stimulation and reaching maximal levels between 60 and 90 min. At Cxcl11 the two upstream transcripts tested appeared even faster, peaking between 30 and 60 min, to be then rapidly downregulated (Figure 2B). In both cases, however, kinetics of induction of upstream extragenic transcription preceded the appearance of the mature mRNA generated from the downstream coding genes, a concept also supported by the analysis of the nascent transcripts (Figure S2). Moreover, extragenic transcription was downregulated when the coding gene reached its maximal level of expression, a result particularly obvious at Cxcl11. This type of behavior was not specific to these two genes, as it could be detected at several other genes associated with inducible upstream extragenic transcription (Figure 2C). Therefore, extragenic transcription associated with inducible gene expression at these loci displays a clear temporal pattern in which upstream (presumably non-coding) transcription precedes the induction of the downstream protein-coding gene. This kinetic behavior is reminiscent of the relative temporal profiles of non-coding versus coding transcription observed in other systems. At the *fbp1* gene in *S.Pombe*, a rapidly induced, low-level upstream transcription (which is required for chromatin opening at the locus) precedes downstream gene activation and is turned off when the gene is activated [36].

Importantly, all the ncRNAs we detected in this analysis accumulated at very low levels, usually hundreds of folds less than the adjacent coding genes. This may reflect the combination of a low transcription rate (indicated by both the low intensity of both the Pol_II peaks and the nascent transcripts shown in Figure S2) and a high instability of the final product (see below).

### Inducible Upstream Extragenic Transcripts Are Strand-Specific, Poly-Adenylated, Unspliced, and Very Unstable Nuclear Species

**Figure 3:**
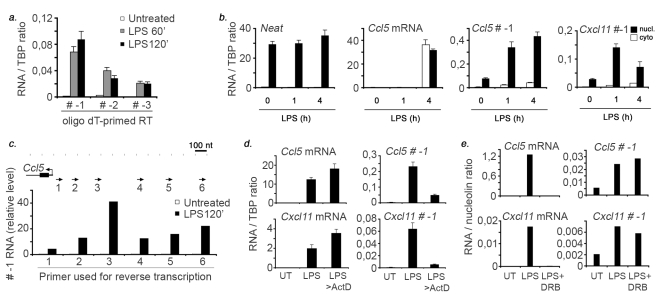
Characterization of the extragenic transcripts generated upstream of LPS-inducible genes. (A) Polyadenylation of extragenic Ccl5 transcripts. Total RNA was reverse-transcribed using oligo-dT primers. cDNA was then amplified with primers corresponding to regions *−1*, *−2*, and *−3* upstream of Ccl5 (as in Figure 1). (B) Upstream extragenic transcripts are nuclear RNAs. Macrophages were fractionated before RNA extraction. RNA from the cytoplasmic and nuclear fractions was then reverse transcribed and amplified with the indicated primers. Neat1 is a nuclear non-coding RNA that was used as a control of the fractionation procedure. (C) Extragenic transcription upstream of Ccl5 generates long unspliced transcripts. RNA was reverse transcribed using antisense primers in the region just upstream of Ccl5 TSS, as indicated. cDNA was then PCR-amplified using primers in the extragenic region *−1* (as in Figure 1A). (D) Extragenic Ccl5 and Cxcl11 transcripts are very unstable. Cells were stimulated with LPS for 2 h, followed by a 30 min actinomycinD (5 µg/ml) chase. mRNAs for Ccl5 and Cxcl11 and the corresponding extragenic transcripts were measured by quantitative PCR. UT, untreated. (E) DRB insensitivity of extragenic Ccl5 and Cxcl11 transcripts. Macrophages were stimulated with LPS for 2 h in the presence or absence of DRB (50 µg/ml), as indicated. UT, untreated.

Detailed structural characterization of these inducible extragenic transcripts is hindered by their very low abundance. Priming the reverse reaction with oligo-dT indicates that transcripts generated upstream of Ccl5 are poly-adenylated (Figure 3A). Moreover, they can be detected exclusively in the nuclear compartment (Figure 3B). Priming the cDNA synthesis with antisense primers located upstream of the 5′ of Ccl5 showed that upstream transcription generates long unspliced RNAs extending for a few kilobases (Figure 3C). However, using the same cDNAs we couldn't obtain Q-PCR signals in peaks further upstream (indicated as −2 and −3 in Figure 1A) (unpublished data). cDNAs primed by multiple oligonucleotides on the opposite strand didn't generate any Q-PCR product (unpublished data), indicating that transcription is strand-specific, occurring on the upper strand toward Ccl5, and as such unlikely to reflect random transcriptional events occurring at open chromatin.

Finally we measured the stability of these transcripts using an actinomycinD chase. In comparison to both the mRNAs generated by the associated protein-coding genes and some known lncRNAs (like Xist and Neat), the upstream non-coding transcripts were very unstable, being reduced by 80% to 90% after a 30 min actinomycinD treatment (indicating a half-life lower than 7.5 min) (Figure 3D and Figure S3). High instability of a subset of lncRNAs both in yeast and mammals mainly depends on degradation by the nuclear exosome [39],[40] and often results in the generation of more stable short RNA products [41], which in principle might be responsible for downstream functional effects.

Another interesting property of some of the upstream transcripts is that, unlike mRNAs, they are poorly sensitive to DRB treatment (Figure 3E). DRB is an inhibitor of Cdk9, the catalytic subunit of the elongation factor pTEFb [42]. Cdk9 acts on multiple substrates to promote Pol_II entry into the elongation phase and cotranscriptional mRNA processing. The previous finding that up to 40% of nuclear RNA synthesis is unaffected by DRB treatment, as opposed to the 95% reduction of cytoplasmic polyadenylated transcripts [43], may indirectly suggest that at least part of extragenic transcription is subjected to control mechanisms different from those acting at protein coding genes, and specifically that P-TEFb may not be required for Pol_II activity at some of these regions.

### Genome-Wide Annotation of Extragenic Pol_II Transcription Sites

Browsing through the data indicated some major challenges towards a systematic and correct identification of extragenic Pol_II peaks. The most obvious one was represented by the extension of elongating Pol_II molecules several kilobases beyond the end of annotated protein coding genes, namely in regions that by definition are extragenic. This is most likely due to the lack of specific and strong termination signals for RNA Pol_II. Moreover, alternative TSSs located upstream of the annotated ones contribute to create ambiguity in extragenic Pol_II peak annotation. To systematically annotate sites of extragenic transcription, we first filtered out all Pol_II signals overlapping UCSC known genes as well as peaks within 10 kb from the 3′ end of annotated genes (which after several tests proved to be an optimal length to eliminate most signals due to Pol_II tracking from the upstream gene). It is important to stress that because of this design, our analysis does not take into account gene boundaries, which represent a major source of long and short non-coding RNAs [40],[41],[44]–[47].

**Figure 4:**
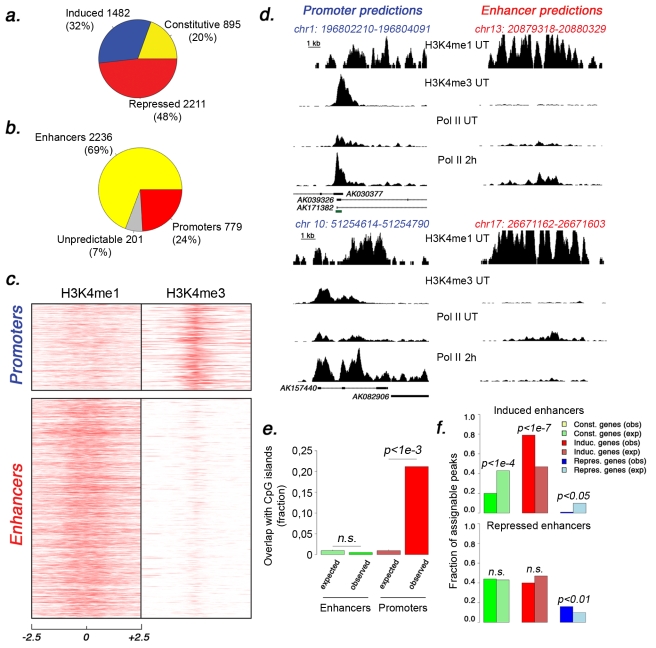
Identification of enhancer-associated and promoter-associated extragenic Pol_II transcription sites. (A) Pie chart showing the three groups of extragenic Pol_II peaks (classified on the basis of Pol_II changes after stimulation) in untreated and LPS+γIFN-treated macrophages. Numbers refer to Pol_II peaks before SVM classification, clusterization, and filtering against Ensembl protein-coding genes. (B) The pie chart shows the results of the machine-learning approach used to classify extragenic Pol_II clusters as belonging to promoters or enhancers. Numbers refer to Pol_II peaks after clusterization and filtering against Ensembl protein-coding genes. (C) Enhancer and promoter predictions. Regions of extragenic Pol_II transcription were classified as enhancers or promoters/TSSs using a machine-learning algorithm recognizing alternative H3K4me3/H3K4me1 patterns. Each line represents a 5 kb region centered around the summit of a Pol_II peak (±2.5 kb). Peaks are shown from chromosome 1 (top) to chromosome X (bottom). (D) Examples of predicted promoters and enhancers. ChIP-Seq profiles at regions containing representative extragenic transcription sites belonging to the two groups are shown. The coordinates indicate the position of the Pol_II peak. The green square indicates a CpG island. (E) Association of predicted enhancers and promoters with CpG islands. Expected and observed fractions are shown. (F) Correlation between LPS-induced Pol_II changes at predicted transcribed enhancers and at the neighboring protein-coding gene. Inducible enhancers (upper panel) and repressed enhancers (lower panel) are shown. Observed (obs) and expected (exp) fractions for each group of genes (constitutive, repressed, and inducible genes) are shown together with the respective *p* value (the *p* value may refer to either an over- or an under-representation). Expected fractions were calculated on the basis of the relative frequency of each group (constitutive, induced, repressed genes) with respect to all Pol_II positive genes. n.s., non-significant.

The initial list was eventually curated for additional filtering (mainly to eliminate Pol_II signals showing continuity with upstream genes), leading to 4,588 high-confidence extragenic Pol_II peaks. Using a statistical approach for ChIP-Seq data analysis [48] we classified these peaks as constitutive (895), inducible (1,482), or repressed (2,211) in response to stimulation (Figure 4A and Table S1).

### Classification of Extragenic Pol_II Sites Based on Chromatin Signatures

Chromatin signatures generated by specific combinations of post-translational modifications of core histone tails are powerful and sensitive indicators of functionality [49]–[51]. A simple, yet informative combination of modifications includes the mono-methylation of H3K4 (H3K4me1) and the tri-methylation of the same residue (H3K4me3). TSSs of genes that are either active or poised for activity are characterized by high levels of H3K4me3 (peaking just downstream of the TSS and confined to a few nucleosomes), flanked on both sides by regions enriched for H3K4me1. Conversely, enhancers display high levels of H3K4me1, usually distributed over several kilobases, associated with low or no H3K4me3 (H3K4me1^hi^/H3K4me3^lo^ domains) [52],[53]. Enhancers are also frequently bound by the histone acetyltransferase p300 [52].

In order to assign the extragenic Pol_II clusters in our dataset to either TSSs of lncRNA genes or to enhancers, we used a machine-learning algorithm (Text S1). The algorithm was instructed to discriminate enhancers from promoters using the H3K4me3/H3K4me1 chromatin profiles at 556 informative (unambiguous) extragenic p300 peaks (described in [54]) and the H3K4me3/H3K4me1 profiles at an identical number of promoters/TSSs with a broad range of Pol_II levels. This approach was validated by multiple tests (see Text S1) including its ability to properly classify ChIP-Seq peaks of the macrophage TF PU.1, which we found to be strongly but not exclusively enriched in enhancers [54]: PU.1 peaks that were classified as promoters/TSSs using this algorithm overlapped annotated TSSs of UCSC known genes in 67% of cases, while PU.1 peaks classified as enhancers overlapped annotated TSS only in 7% of cases (and in most cases visual inspection confirmed that these TSS did not show a typical signature of promoters).

### A Large Fraction of Extragenic Pol_II Activity Occurs at Enhancers

We thus applied this machine-learning algorithm to the dataset of 4,588 extragenic Pol_II peaks described above. We found that 3,227/4,588 peaks were contained in regions with a chromatin signature of enhancers, 1,004 were in regions with a signature of active or poised TSSs, while 357 were associated with regions with a non-predictive signature. Peaks were then clustered (see Methods) and then filtered against Ensembl protein coding genes to definitively discard regions with protein-coding potential. The final dataset consisted of 3,216 Pol_II clusters, including 2,236 enhancers (69%), 779 promoters (24%), and 201 unpredictable regions (7%) (Figure 4B and Table S4). Chromatin signatures at the enhancer and promoter groups are shown in Figure 4C, and examples of predicted enhancers and promoters associated with extragenic Pol_II clusters are shown in Figure 4D. The chromatin signature at the region upstream of Ccl5 is also compatible with its enhancer activity (Figure S4). If these predictions are correct, an obvious expectation is that the group associated with the promoter/TSS signature should be enriched for CpG islands. This was indeed the case: 165/779 promoters (21.2%) were associated with an underlying CpG island (*p*<1e-3) as compared to only 11/2,236 enhancer clusters (0.5%, which is similar to what was found in random sets of genomic sequences with similar composition) (Figure 4E). The association between putative ncRNA genes and CpG islands is clearly much lower than observed at protein-coding genes (72%) [55]; however, our results are similar to those reported by Ponjavic et al. for ncRNA genes expressed in mouse development, which were associated with CpG islands in about 30% of cases [56]. The TSSs of annotated, bona-fide RNA genes (like Neat1, Malat, and Xist) [2] have chromatin features analogous to those of protein-coding genes and perfectly fitting the pattern of our promoters/TSSs group (Figure S5 and unpublished data). This is in keeping with the notion that lncRNA genes can be retrieved using the same H3K4me3/H3K36me3 chromatin signature that was originally described at active protein coding genes [25].

We next investigated the relationship between the transcriptional activity of predicted enhancers and that of the associated protein-coding genes. First, we assigned predicted transcribed enhancers to adjacent coding genes if distant from them less than 20 kb. We considered this restrictive criterion essential to limit incorrect or arbitrary matches. Enhancers whose association with Pol_II was induced or increased by stimulation were strongly associated with inducible genes (*p*<1e-7 when compared to the expected fraction), while association with constitutive and repressed genes was underrepresented in a statistically significant manner (Figure 4F and Table S5). In a specular manner, repressed enhancers were associated with repressed genes, albeit at low statistical significance (Figure 4F). It should be stressed that repressed enhancers are also associated with a large number of genes that are induced by stimulation. Although from a statistical point of view this group of inducible genes is underrepresented as compared to what is expected, the possibility should not be discounted that transcriptional downregulation of an enhancer may be involved in the activation of the associated gene, possibly by relieving transcriptional interference [29].

### Evidence for Active Transcription at Enhancers

In the cases shown in Figure 2 we could detect and measure low-abundance long RNAs (≥200 nt) generated at regions of extragenic Pol_II binding. However, Pol_II recruitment to chromatin is not necessarily followed by elongation [57],[58]. To address this crucial issue, we carried out several complementary analyses and experiments. First, we analyzed the overlap between extragenic Pol_II sites and annotated ncRNAs datasets. We used two different catalogues: a “macroRNA” dataset (2,168 ncRNAs) generated by the FANTOM consortium by massive cDNA sequencing [26] and then filtered to eliminate RNAs overlapping all current protein-coding gene annotations [24],[59], and a dataset of large intervening ncRNAs (1,408 “lincRNAs”) identified by the H3K4me3/H3K36me3 chromatin signatures characteristic of bona fide active genes [25] and then filtered against the Ensemble protein-coding genes (Table S2). These two catalogues show little overlap, suggesting that each of them includes only a small fraction of a presumably much larger ncRNA repertoire [59]. 26/2,236 predicted enhancers and 21/779 promoters/TSSs overlapped annotated macroRNAs (albeit low, the overlap was statistically significant) (Table S3). LincRNAs were associated with the promoter group (122/779; 15.6%) and, to a lower extent, to the enhancer group (167/2,236; 7.4%) (Table S3). As lincRNAs were identified on the basis of an H3K4me3/H3K36me3 chromatin signature that distinguishes active genes, the overlap with the enhancer group may appear surprising. However, visual inspection of these enhancers was consistent with the notion that they represent regulatory regions located within these extended H3K4me3/H3K36me3 domains (see Figure S6).

**Figure 5:**
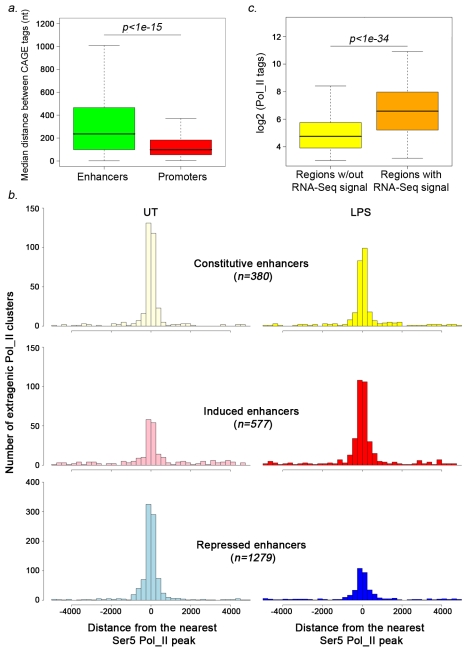
Evidence for active transcription at Pol_II-associated enhancers. (A) Distribution of the median distance between CAGE tags clusters overlapping the regions predicted as either enhancers or promoters. (B) Correlation between total and active Pol_II (phosphorylated at Ser5 of the CTD) at enhancers. The graphs illustrate the distance between extragenic Pol_II peaks predicted as enhancers and the closest P-Ser5 Pol_II peak. (C) Extragenic regions associated with RNA_Seq signals display higher Pol_II occupancy than those without RNA-Seq signals.

Second, using a database of CAGE tags generated from the FANTOM consortium [60], we found that the transcriptional potential of 72% of regions in the promoter group and 53% in the enhancer group was supported by overlapping CAGE tags. In interpreting these data it should be considered that the lncRNAs generated at the β-globin LCR do not contain a CAP at their 5′ end [61], which implies that a fraction of the transcripts generated at regulatory regions is not represented in CAGE tags libraries. Interestingly, the median distance between multiple CAGE tags is significantly higher in enhancers than in promoters (Figure 5A). These data confirm the transcriptional potential of predicted enhancers and suggest that while TSSs are tightly clustered in the promoter group, they are distributed over broader distances in the enhancer group (presumably generating primary transcripts with heterogeneous 5′ ends).

Third, we generated ChIP-Seq datasets in untreated and LPS-treated macrophages using an antibody that recognizes the large Pol_II subunit Rbp1 only when phosphorylated at Ser5 of its C-terminal domain (CTD). Ser5 phosphorylation by TFIIH occurs at the transition to transcription initiation and is maintained throughout the length of transcribed genes to be then removed by a phosphatase at the very 3′ end [38]. Ser5-P was extensively associated with both predicted enhancers and promoters in our datasets (Figure 5B, Table S6). Median Ser5 peak length is 479 bp, with a minimum of 110 bp and a maximum of 7341 bp, indirectly suggesting that in most cases long (>200 nt) primary transcripts are generated. This result confirms that, independently of the final abundance of the transcripts, enhancers associated with Pol_II are actively transcribed. Similar results were obtained for promoters (Figure S7).

Fourth, we analyzed by quantitative RT-PCR a representative set of 100 predicted enhancers within the whole range of *p* values associated with the corresponding Pol_II peaks (as in Table S1). Primers were designed to generate 200 nt amplicons. 96/100 tested regions generated detectable transcripts (Table S7), indirectly indicating that the vast majority of extragenic Pol_II peaks likely generate transcripts.

Due to their very low abundance, a comprehensive analysis of extragenic ncRNAs and their detailed structural characterization present obvious difficulties. RNA sequencing is a powerful approach for detection of potentially all RNA species in a cell, although low abundance transcripts can be identified only at very high sequencing depth. As an initial step toward characterization of enhancer-associated transcripts, we generated an RNA-Seq dataset in untreated macrophages using total nuclear RNAs. At the level of sequencing depth we reached (11.5 million aligned tags from four Solexa GAII lanes) we could detect 225,439 transcripts corresponding to 13,702 RefSeq genes and 28,247 UCSC known genes. We found RNA-Seq tags overlapping 193/484 promoters and 369/1,660 enhancers active in untreated macrophages (corresponding to the constitutive and repressed groups; *p*<1e-3 compared to random sets of intergenic genomic sequences). In most cases, however, low density of tags precluded the identification of well-defined transcripts. Importantly, the extragenic regions associated with RNA-Seq tags displayed median Pol_II signals about 1.5 orders of magnitude higher than the regions for which transcripts could not be detected at this sequencing depth (Figure 5C). Therefore, only the transcripts produced at the extragenic regions with high transcriptional activity could be detected (Table S4). Nevertheless, these data further confirm that Pol_II-bound extragenic regions are in general subjected to active transcription.

### Evidence of Functionality of Enhancer-Type Extragenic Transcribed Regions

**Figure 6:**
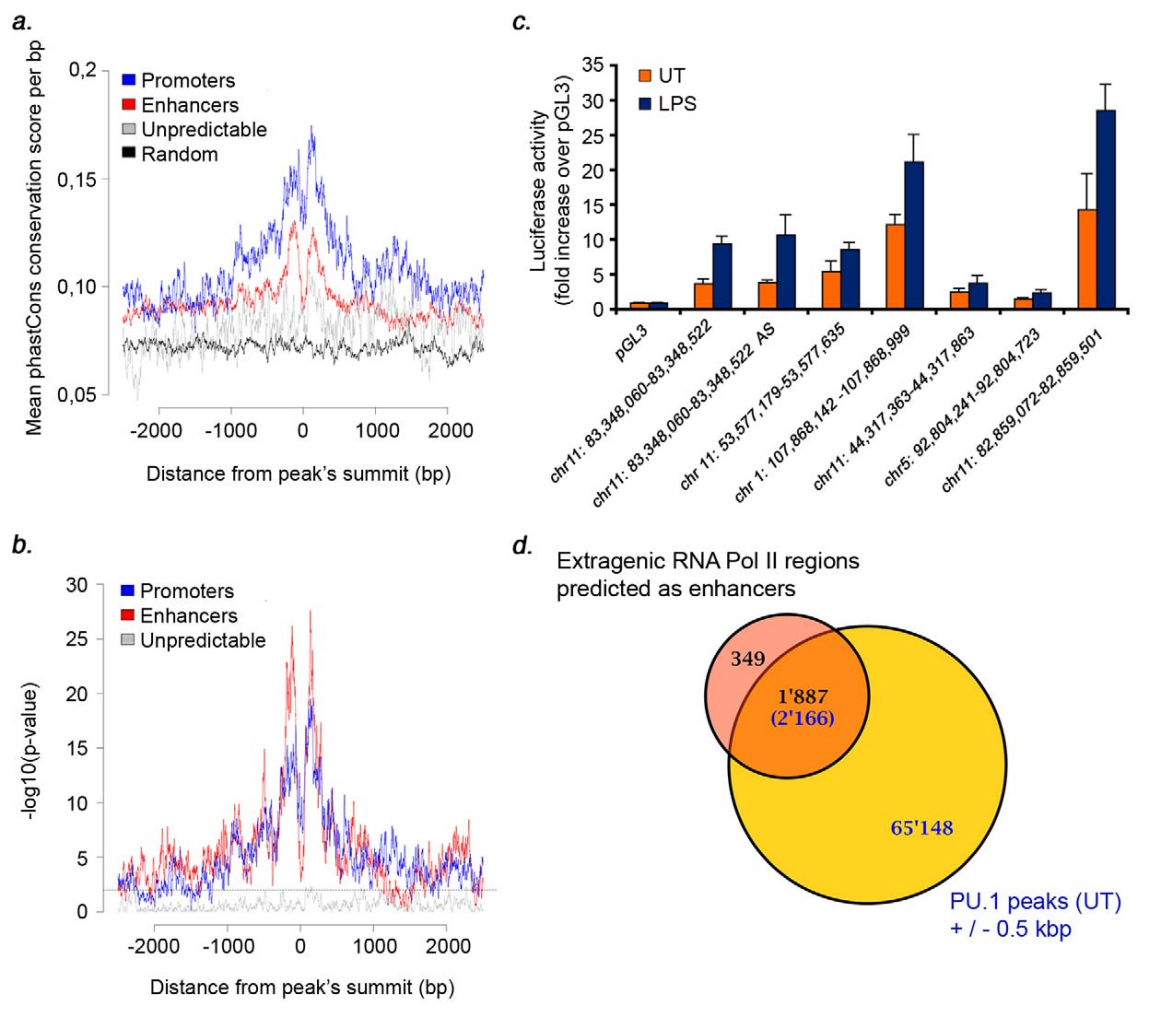
Signatures of functionality at enhancer-associated extragenic transcription sites. (A) Sequence conservation at extragenic Pol_II transcription sites. Average conservation scores (phastCons score per bp) in the enhancer, promoter, and unpredictable groups are shown. Pol_II peaks were centered around their summit. (B) Statistical significance of sequence conservation in the three groups was evaluated as compared to random sets. The *y*-axis indicates the *p* value of the deviation from random. The horizontal grey line indicates the threshold for statistical significance (set to *p*<0.01). (C) Functional evaluation of predicted enhancers in reporter assays. The indicated regions were subcloned in the pGL3 promoter vector, which bears a minimal promoter, and transfected in Raw264.7 macrophage cells. Cells were stimulated with LPS for 16 h before harvesting. Errors bars, S.D. (D) Overlap of extragenic transcription sites with an enhancer-associated chromatin signature with experimentally determined binding sites of the hematopoietic transcription factor PU.1. PU.1 peaks ± 500 bp (identified in a ChIP-Seq experiment in untreated macrophages) were considered. Black numbers refer to Pol_II clusters, while blue numbers refer to PU.1 peaks.

While a large fraction of extragenic transcription sites bear an enhancer-associated chromatin signature, this doesn't demonstrate that these regions have functional properties of enhancers. We first searched the predicted enhancers for evolutionary signatures of functionality and specifically for evidence of purifying selection. We used phastCons scores in placental mammals [62] to measure the degree of conservation in the three groups of extragenic Pol_II clusters. Both promoters and enhancers were strongly conserved, with overall higher scores in the promoter group (Figure 6A). In both groups conservation was statistically significant as compared to matched random sequence sets (Figure 6B). Conversely, the group of Pol_II clusters with a non-informative chromatin signature did not significantly deviate from random sets.

Sequence conservation in both the enhancer and the promoter group was stronger in the central regions (and precisely in the sequences just flanking the summit of the Pol_II peaks) and it was progressively diluted moving outwards.

We next cloned some of these predicted enhancer sequences in a plasmid bearing a minimal promoter driving luciferase expression and tested their ability to increase reporter gene activity. All the sequences tested increased basal expression and some provided responsiveness to LPS stimulation (Figure 6C). The first sequence from the left was also assayed for orientation-independence of enhancer activity (Figure 6C). As additional evidence that these regions are in fact bona fide enhancers, we tested their ability to fold onto the neighboring promoter using chromosome conformation capture (3C) [63]. The transcribed regions upstream of Ccl5 and Cxcl11 were in fact both associated with the regions surrounding the respective TSS (Figure S8). Association was not dependent on stimulation as it could be found also in basal conditions. In fact, stimulation reduced to a various extent the degree of looping.

Finally, we evaluated the degree of overlap between extragenic Pol_II and binding of the transcription factor PU.1, which (in addition to being recruited to active promoters) is very extensively associated with enhancers in macrophages [54]. Considering a search space of ±500 nt surrounding ChIP-Seq PU.1 peaks, we found that 84.4% of enhancer-type extragenic Pol_II clusters were associated with PU.1 binding (Figure 6D; see Figures S4 and S6 for some examples). PU.1 association with promoter/TSS-type transcribed regions was also very frequent (69.3%), while Pol_II peaks with a non-predictive chromatin signature were associated with PU.1 only in 33.8% of cases. Such a substantial association between extragenic Pol_II and binding of a sequence-specific TF (72% overlap considering the entire dataset) strongly argues against the notion that this extensive transcriptional activity is mere noise and conversely confirms its nature as a regulated process.

### Different Sets of Transcription Factors Are Associated with Different Behaviors of Enhancer-Associated Extragenic RNA Pol_II

Enhancer functionality depends on the transcription factor binding sites (TFBS) contained in their sequence. TFs activated by stimulation with LPS+IFNγ include NF-kB/Rel family members [64], IRFs (interferon regulatory factors) [65], and STAT1 [66]. Moreover, the hematopoietic Ets family member PU.1, which is constitutively expressed at highest levels in macrophages, is highly enriched in enhancers, where it provides context dependence to responses driven by inflammatory TFs [54]. We therefore searched our dataset of 2,236 predicted enhancers associated with extragenic Pol_II for enriched TFBSs. To this aim, we first assembled a library of 338 position weight matrices (PWMs) by combining the DNA binding motifs in the Jaspar database [67] and those in a recently reported set of PWMs for 104 mouse transcription factors [68]. Then we divided the enhancers in three groups based on Pol_II behavior (constitutive, inducible, and repressed) and used a statistical approach [69] to score TFBS enrichment in each group relative to two background sets (namely the whole mouse chr 19 and a set of all 5 kb sequences located upstream of the TSSs of mouse RefSeq genes).

**Figure 7:**
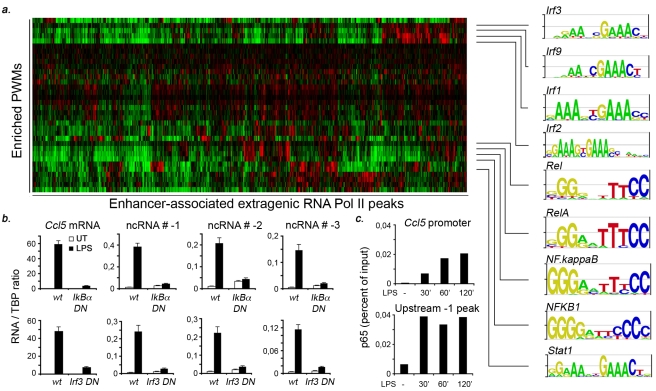
Enrichment of transcription factor binding sites in enhancer-type extragenic Pol_II transcription sites. (A) TFBSs enriched in the set of inducible, enhancer-type extragenic transcription sites. Enrichment was evaluated using two reference datasets (see Methods). Each vertical column in the heat-plot represents a Pol_II peak, while each row corresponds to an enriched PWM. Data are shown after hierarchical clustering. Selected enriched PWMs for inflammatory TFs (IRFs, STAT1, NF-kB) are shown on the right. Increasing red color represents increasing probabilities for a PWM to have a match in the region as compared to randomized sequences with the same nucleotide composition [69]. (B) IRF3 and NF-kB are required for extragenic transcription upstream of Ccl5. Raw264.7 cell lines constitutively expressing a dominant negative Irf3 (IRF3DN) or a general inhibitor of NF-kB (IkBα super-repressor, IkBαDN) were stimulated with LPS as indicated and Ccl5 mRNA or upstream extragenic transcripts were measured by RT Q-PCR. (C) Binding of NF-kB to the Ccl5 promoter and to a region corresponding to the *−1* Pol_II peak. NF-kB binding was measured using an anti-p65 ChIP.

In the inducible group we found a strong enrichment for IRFs and STAT1 (which bind related sites and were recognized by five distinct PWMs), as well as for NF-kB/Rel (identified by four PWMs) (Figure 7A and Table S8). Moreover, the dataset was strongly enriched for PU.1/Spi1 PWMs, which is in keeping with its association with enhancers [54]. The constitutive group, in addition to a strong enrichment for PU.1/Spi1, showed a comparatively lower but anyway significant enrichment for IRF/STAT1 and NF-kB/Rel PWMs (Table S8). In this regard, it should be noticed that some of the enhancers that we define as “constitutive,” in fact show LPS-induced increases in Pol_II levels that do not reach the threshold we set for the inclusion among the inducible peaks. Remarkably, the group of putative enhancers repressed by stimulation was strongly enriched for PU.1/Spi1 but not for any of the PWMs for the inducible, inflammatory TFs associated with the other two groups (Table S8). Therefore the enhancers whose association with Pol_II is reduced by stimulation appear to represent a distinct group with a completely different TFBS composition. Importantly, also the group of the induced promoters (and to a lesser extent the one including the constitutive promoters) was enriched for binding sites for inflammatory TFs (Table S8), indicating that the TFs driving the inflammatory gene expression program also control many canonical RNA genes.

We next evaluated if the identified TFBSs are functional. Some of the inducible Pol_II peaks in the region upstream of Ccl5 scored positively for IRF3 (as well as other IRFs) and NF-kB, which are known to coregulate Ccl5 expression [70]. Blocking IRF3 and NF-kB activity with specific mutants in stable Raw264.7 macrophage cell lines (kindly provided by G. Cheng, UCLA) blocked not only the induction of the Ccl5 mRNA but also the appearance of the upstream non-coding transcripts (Figure 7B). Moreover, the NF-kB subunit p65/RelA was recruited to the Ccl5 upstream region (Figure 7C), thus further supporting the functionality of the identified sites. Interestingly, maximal p65 recruitment to this region preceded recruitment to the NF-kB binding sites contained in the Ccl5 promoter, which is in keeping with the faster kinetics of induction of upstream transcription as compared to that of the Ccl5 mRNA (as shown in Figure 2).

### Enhancer Associated Extragenic Transcription May Promote Domain-Wide Acetylation and Pol_II Loading on Downstream TSSs

**Figure 8:**
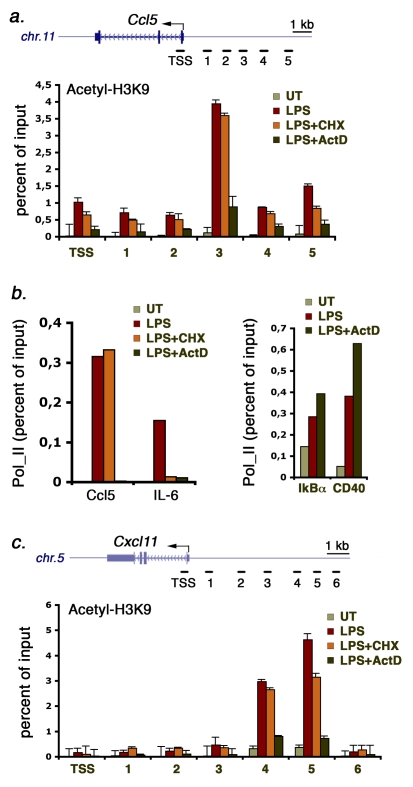
Functional consequences of transcriptional inhibition on extragenic histone acetylation at the Ccl5 and Cxcl11 loci. (A) H3K9 acetylation at the transcribed region (about 5 kb) upstream of Ccl5 was measured by ChIP in the absence or presence of CHX (10 µg/ml) or ActD (5 µg/ml) as indicated. UT, untreated macrophages. Position of the amplicons (transcription start site [TSS] and five extragenic amplicons indicated by progressive numbers) is indicated. (B) Left panel: inhibition of transcription but not translation blocks the activation of the primary response gene Ccl5 [73]. Conversely, Pol_II recruitment to the TSS of the secondary gene IL-6 is equally sensitive to CHX and ActD. Right panel: ActD does not inhibit Pol_II recruitment to IkBα and CD40. (C) Transcription is required for inducible H3K9 hyperacetylation at the transcribed region upstream of Cxcl11. The position of amplicons at the TSS and upstream of the gene are indicated.

As we could detect thousands of enhancer-associated extragenic Pol_II peaks with distinct behaviors, some degree of functional heterogeneity is expected. Moreover, definitive understanding of the function of each extragenic transcription site would require dedicated genetic approaches to interfere with Pol_II loading and/or elongation (like the knock-in of transcriptional terminator sequences; see for instance [11],[36]). Attempts to deplete ncRNA generated at enhancers by RNAi were not successful, which likely reflects their constitutive instability (see Discussion). We tried however to get an initial glimpse into the functional impact of transcription through enhancers in this system. One model supported by experimental data is that extragenic transcription leads to the repeated passage of several Pol_II-associated enzymes, including Swi/Snf remodeling complexes [71],[72] and histone acetyltransferases [35], through chromatin regions, thus leading to extensive remodeling and changes in accessibility [32]. We first found that macrophage activation is associated with a domain-wide increase in acetylation at the transcribed regions upstream of Ccl5 (Figure 8A and unpublished data). Domain-wide hyperacetylation was strongly reduced by treatment with actinomycinD but not with the protein synthesis inhibitor cycloheximide (CHX). Importantly, Ccl5 is a primary response gene and as such it is not sensitive to CHX treatment [73]. Therefore, while new protein synthesis does not impact on acetylation of the locus, new transcription is required for maximal acetylation both at the TSS and at upstream regions. ActD (but not CHX) treatment also prevented recruitment of Pol_II at the Ccl5 TSS (Figure 8B, left). Conversely, at a secondary gene (interleukin 6, IL-6), both CHX and ActD completely blocked Pol_II recruitment (Figure 8B, left). The effects of ActD on Pol_II recruitment to TSSs were not general, as they could not be detected at two other genes tested (Figure 8B, right). Therefore, with all the due cautions required in experiments with global inhibitors, it seems that the act (or the products) of transcription (rather than the induction of new protein products) is involved both in acetylation through the Ccl5 locus and in gene induction.

A similar behavior was found at the Cxcl11 upstream regions (Figure 8C). Here we could detect a high basal level of acetylation in the regions corresponding to the extragenic Pol_II peaks. Acetylation was strongly increased by stimulation and returned to basal levels upon ActD (but not CHX) treatment, thus indicating that also in this case extragenic transcription (or its products) may be involved in controlling the chromatin state of the locus.

## Discussion

The main finding of this study is that RNA Pol_II association with, and productive transcription of, a subset of *cis*-regulatory regions accounts for a sizeable fraction of transcription sites located outside of coding gene borders. It is important to notice that the design of our study—which is based on the analysis of Pol_II occupancy in regions not overlapping annotated protein-coding genes—implies that gene boundaries, which contribute in a substantial manner to the repertoire of short and long ncRNAs in mammalian cells [40],[41],[44]–[47], were not taken into consideration.

The concept that enhancers and LCRs in some cases undergo transcription was previously demonstrated at individual loci in various experimental models [9]–[11],[29],[30],[36]. Our data demonstrate on a genomic scale that this is a common occurrence. However, based on our data on enhancers in this specific system [54], as well as reports in other models [53], it seems clear that non-transcribed enhancers (in the order of magnitude of dozens of thousands in every given cell type) greatly outnumber the transcribed ones, which raises some obvious questions.

First, can enhancers be classified on the basis of being transcribed or not, and do Pol_II-transcribed enhancers represent a functionally and mechanistically homogeneous group? A simple model, compatible with a large body of experimental data, is that functionality of transcribed enhancers and LCRs indeed depends on the directional movement of Pol_II along their sequence [32]. Large chromatin domains often undergo regulated and extensive modifications (like acetylation and reduction of nucleosomal density) controlling their accessibility and functionality: in such cases it is difficult to imagine how chromatin-modifying enzymes recruited to discrete sites by association with sequence-specific TFs can promote such large scale changes. Conversely, loading the same enzymes onto elongating Pol_II complexes provides a regulated and specific way to catalyze rapid changes across extended regions, thus establishing transcriptional competence (discussed in [32]). An example of a specific effect of the transcription process itself, in which the ncRNA product apparently has no direct role, is provided by the *PHO5* gene in yeast, whose activation requires nucleosome eviction stimulated by non-coding transcription across its promoter [31]. When the level of the ensuing ncRNA was artificially increased (by either overexpression or by inactivation of the nuclear exosome), no consequences on nucleosome depletion were found [31]. In other cases it was shown that the ncRNAs generated from regulatory regions is functional [8], either by controlling the deposition of epigenetic modifications [21] or by promoting the recruitment [20] or stimulating the activity [12] of transcriptional activators. In some of these cases, it is implicit that the ncRNAs would act at the production site, possibly when still associated with elongating Pol_II. This model may well apply to ncRNAs generated at enhancers, whose function may relate to the control of local chromatin features. Overall, the role of ncRNA transcripts versus transcription in conveying regulatory information likely varies depending on the regulatory region considered, and ad hoc experiments will be required to understand the relative frequency of the two groups of mechanisms. For those enhancers whose associated transcripts will be demonstrated to be functional (as in [20]), their distinction from canonical RNA genes may appear conceptually subtle and in the end rely exclusively on their distinct chromatin signature. However, we believe that an additional important aspect should be considered in distinguishing enhancers that generate functional RNAs from canonical RNA genes: the *local* and *temporally restricted cis*-regulatory role of the enhancer-associated ncRNA (temporal restriction being related to the rapid degradation of these transcripts after they are synthesized). On the contrary, ncRNAs generated from canonical RNA genes in most cases act at a distance from the production site (e.g. Neat1) [2]; even when acting in *cis*, as in the case of Xist and Air, they coat (and functionally affect) broad chromosomal regions, thus in fact exerting an activity that extends far beyond the borders of their site of synthesis.

In this context, it appears very relevant to bring into focus the conceptual and technical problems related to the mechanistic dissection of the ncRNAs generated at regulatory regions. Assessing the functionality of these ncRNAs will require that their specific elimination or depletion be dissociated from any effect on the underlying transcription. Therefore, knocking-in transcriptional terminators to interfere with Pol_II elongation (see for instance [11]) is in fact non-informative in this regard. Depletion of ncRNAs by RNAi efficiently works when applied to stable transcripts encoded by RNA genes [19]. However, enhancer-generated transcripts are very unstable, possibly due to a constitutive surveillance by the nuclear exosome [39],[40], leading to their complete degradation or the generation of short RNAs [41]. Moreover, the role of nascent ncRNAs in targeting to chromatin specific regulators with RNA binding modules (as suggested in [20]) may be limited to a very short window of opportunity during which proximity to chromatin is maintained, namely the time of Pol_II passage over a specific genomic region. Low level of expression of these ncRNAs (see Figure 2) may reflect the restriction of their activity to the genomic regions where they are synthesized. For both reasons, reducing their levels by RNA interference (before they are degraded or before they exert a local and transient functional activity) may not be feasible, at least using simple tools. On the other hand, for those ncRNAs acting at their site of production, overexpressing them cannot recapitulate their normal function.

A second outstanding question pertains to the identity of the determinants of enhancer association with RNA Pol_II factories and of the molecular mechanisms controlling transcriptional initiation at these regulatory regions. It seems clear that in some cases Pol_II can be loaded at multiple positions along the enhancer/LCR [61], a result in keeping with the presence of multiple distant CAGE tag clusters at enhancer regions in our dataset (Figure 5C). Still, the directionality of transcription (see also Figure 3C) implies a tight control upon formation of the preinitiation complex and rules out the possibility that transcription is a mere consequence of random Pol_II collisions with accessible loci.

A third related question is whether enhancer-associated transcription is mechanistically different from transcription of protein- and RNA-coding genes. This possibility is supported by several observations, including the resistance to the general elongation inhibitor DRB of part of nuclear transcription ([43] and our own data) and, as discussed above, the fact that enhancer associated transcription often initiates at multiple points along the sequence of the enhancer [61], as if rules for initiation were less stringent at enhancers as compared to protein and RNA genes.

One important aspect of extragenic transcription, and particularly the fraction not associated with putative RNA-coding genes bearing a promoter signature at their 5′ end, is that it should be unambiguously distinguished from the transcriptional noise that may arise from spontaneous collisions of the Pol_II transcriptional machinery with some genomic sequences [7],[8]. A form of noise that has been recently described is represented by waves of transcription extending from highly active immediate early genes (IEGs) into neighboring sequences, including genes and intergenic regions [14]. This “ripple effect” is somehow similar to the inducible extragenic transcription we show here, and therefore it deserves a careful analysis. The interpretation of the authors [14] is that ripples start from IEGs and extend into the adjacent regions: because of this behavior these Pol_II waves should be considered noise, and specifically the downstream consequence of a strong gene activation that cannot be confined to the limits of the gene itself. It should be noticed that in the system used by Ebisuya et al., namely growth factor stimulation of fibroblasts, IEG induction is extremely fast, with Pol_II peaking in several cases at 10 min after stimulation. Therefore, this system offers limited possibilities to identify complex temporal sequences in the activation of upstream extragenic regions versus associated coding genes. Conversely, the system used in this study has the advantage that genes are induced in a kinetically complex fashion [73],[74], in some cases relatively long after the initial stimulation. At genes like Ccl5 and Cxcl11, as well as at several others (see Figure 2), this kinetic behavior allowed us to identify a recurring temporal pattern in which upstream non-coding transcription not only precedes the induction of the neighboring protein-coding gene but also peaks when the activity of the associated coding gene is hardly detectable (which is similar to what was described at the inducible *fbp1* gene in yeast) [36]. Conversely, a ripple effect should parallel RNA Pol_II activity at the associated coding gene and reach maximal levels when the coding gene is at its peak of activity. A second expected feature of a ripple effect is that extragenic waves of Pol_II should show continuity with Pol_II elongating from the inducible coding gene. In our dataset, this is an unusual occurrence (see Figures 1 and 2 and Figure S1 for examples).

The direct evidence arguing against the possibility that extragenic Pol_II reflects transcriptional noise comes from four groups of data we obtained in this study: (1) the presence of an enhancer-associated chromatin signature [52], (2) the enrichment for inflammatory TFBSs like NF-kB and the IRFs, (3) the functionality of some tested regions in heterologous reporter assays, and most importantly, (4) the very extensive overlap between sites of extragenic transcription and binding sites for the TF PU.1, which is required for macrophage differentiation [75]–[77] and function [78],[79], and very extensively marks enhancers [54]. The only group of extragenic Pol_II peaks (about 8% of the peaks in the dataset) that in principle may represent noise (although it is not possible to formally demonstrate it with our analysis) is the one consisting of regions without an informative chromatin signature: in fact, this group shows levels of sequence conservation that are not significantly different from those of random sequences (see Figure 6B). Overall, we can safely conclude that transcribed extragenic regions with an enhancer-associated chromatin signature represent in most cases sites of highly regulated Pol_II recruitment and elongation, possibly relevant for their function as enhancers.

An additional aspect worthy of attention is that at least in this system extragenic Pol_II peaks are more frequently repressed than induced by stimulation. While in many cases repression correlated with downregulation of the associated genes, in several others it correlated with gene activation of a neighboring gene. A reasonable hypothesis in this case is that, similar to what was described in other models [29],[30], extragenic transcription extending into neighboring genes may interfere with their activity: therefore gene induction can occur only when transcription from adjacent extragenic regions is switched off.

In conclusion, our study demonstrates that the pervasive transcription occurring in mammalian genomes [1] is contributed not only by RNA-coding genes but also by a large number of enhancers associated with constitutive or regulated Pol_II transcriptional activity. These data are relevant for functional genomic annotations and at the same time indicate that Pol_II-dependent transcription is integral to the activity of a fraction of functional *cis*-regulatory elements.

## Materials and Methods

### Cell Culture and Materials

Bone marrow cells isolated from female Fvb/Hsd mice were plated in 10 cm plates in 5 ml of BM-medium (high glucose DMEM supplemented with 20% low-endotoxin fetal bovine serum, 30% L929-conditioned medium, 1% glutamine, 1% Pen/Strep, 0.5% Sodium Pyruvate, 0.1% β-mercaptoethanol). Cultures were fed with 2.5 ml of fresh medium every 2 d. Stimulations were carried out at day 7. Raw264.7 were cultured in high glucose DMEM containing 10% low endotoxin FCS. Clones stably expressing dominant negative IRF3 and IkBα super-repressor were a gift of G. Cheng (UCLA) [70]. ActinomycinD, cyclohexymide, and DRB were from Sigma and were used at a final concentration of 5 µg/ml, 10 µg/ml, and 50 µg/ml, respectively. The anti-p65 antibody used in the ChIP in Figure 5C was from Santa Cruz (*sc*-372). The anti-acetylH3K9 antibody used in Figure 6 is from Millipore (#07-352).

### ChIP-Seq Datasets

The RNA Pol_II and H3K4me3 ChIP-Sequencing datasets are described in [37]. The H3K4me1 and PU.1 datasets are described in [54]. Briefly, the RNA Pol_II ChIP-Seq experiment was carried out in unstimulated and LPS+γIFN-stimulated (2 h) macrophages using an antibody recognizing all isoforms of the large Pol_II subunit, Rbp1 (Santa Cruz *sc*-899). The Ser5-Pol II ChIP-Seq datasets were generated using the Ab5131 antibody from Abcam, which recognizes the RNA Pol_II CTD repeat YSPT(phospho)SPS. The H3K4me3, H3K4me1, and PU.1 datasets used in this study were all obtained in unstimulated cells, and antibodies were from Abcam (H3K4me3, Ab8580; H3K4me1, Ab8895) or Santa Cruz (PU.1 *sc*-352). Datasets are available for download from NCBI's Gene Expression Omnibus (GEO, http://www.ncbi.nlm.nih.gov/geo), accession numbers GSE17631, GSE19553, GSE19991.

### RNA Sequencing

Nuclei were isolated as described in the section below and total nuclear RNA was extracted using Trizol. After quality control, RNA was processed following the same standard Solexa protocol recommended for mRNA sequencing. The dataset is available for download from GEO, accession number GSE20370.

### Quantitative RT-PCR and Nascent Transcript Analysis

RNA was extracted from macrophages using Trizol (Invitrogen) and reverse transcribed with random hexamers. In some experiments oligo-dT or gene specific oligonucleotides were used to prime the reverse transcription, as indicated in the text. For isolation of nascent transcripts, cells were lysed in HB buffer (10% glycerol, 60 mM KCl, 15 mM NaCl, 1.5 mM HEPES pH 7.9, 0.5 mM EDTA) containing 0.3 M sucrose and 0.8% NP40. Nuclei were then pelleted through a 0.9 M sucrose cushion in HB buffer and then resuspended in 100 µl of NRB (75 mM NaCl, 20 mM Tris-HCl pH 7.5, 0.5 mM EDTA, 50% glycerol, 100 µg/ml yeast tRNA); lysis was carried out by addition of 750 µl of NLB (0.3 M NaCl, 20 mM HEPES pH 7.6, 0.2 mM EDTA, 7.5 mM MgCl_2_, 1 M urea, 1% NP-40, 100 µg/ml yeast tRNA). Chromatin was then pelleted in microfuge at 4°C and nascent transcripts extracted in Trizol. As control of the lack of genomic DNA contamination, Q-PCR was also carried out on RNA that was not reverse-transcribed. The sequences of the primers used are in Tables S7 and S9.

### Computational Approaches and Data Analysis

Computational procedures, including the machine-learning algorithm used to classify enhancers and promoters, are described in detail in Text S1.

### Transient Transfections and Reporter Assays

RAW264.7 cells were transiently transfected in a 24-well format with 0.8 µg of empty vector (pGL3-promoter vector, Promega) or vectors containing the specified genomic regions (Table S10) with Lipofectamine 2000 (Invitrogen) according to the manufacturer's protocol. Twenty-four h after transfection, cells were treated with LPS (10 ng/ml) and luciferase assay (Bright-Glo, Promega) was performed 16 h after treatment. Values are expressed as fold increase in luciferase counts over the empty vector for each cell line.

## Supporting Information

Figure S1 **Inducible extragenic Pol_II peaks occurring upstream of LPS-inducible genes.** (A) The Cxcl9-Cxcl11 chemokine gene clusters with two Pol_II peaks upstream of Cxcl11 highlighted. (B–D) Three additional representative genomic regions are shown. The extragenic Pol_II peaks are indicated by horizontal lines. Peaks can also be detected at lower levels in unstimulated macrophages.
(0.55 MB TIF)

Figure S2 **Nascent, chromatin associated transcripts at the Ccl5 (top) and Cxcl11 (bottom) loci were measured in LPS+γIFN-stimulated cells as indicated.** The amplicons indicated are: TSS (transcription start site); A and B (corresponding to two regions contained within the −1 peak in Figure 1 [for Ccl5] and the −1 peaks in Figure S1A [for Cxcl11]). Pol_II ChIP-Seq data in the same regions (2h LPS+γIFN stimulation) are also shown. Error bars, s.e.m.
(0.25 MB TIF)

Figure S3 **Stability of representative RNAs originating from extragenic Pol_II transcription sites.** (**A**) Macrophages were stimulated with LPS for 2 h and then treated for 30 min with actinomycinD (ActD). Stability of the upstream non-coding transcripts is compared to that of the neighboring protein-coding gene. At each panel the *y*-axis on the left (in red) indicates the mRNA levels relative to those of the housekeeping gene TBP, while the *y*-axis on the right (light blue) indicates the levels of the neighboring upstream RNA generated by extragenic transcription. (B) Stability of annotated ncRNAs, including Neat, Xist, two Fantom transcripts, and two Linc RNAs.
(0.61 MB TIF)

Figure S4 **An enhancer-associated chromatin signature in the transcribed region upstream of Ccl5.** The three main sites of extragenic transcription are indicated by shaded blue boxes. The two tracks at the bottom show the ChIP-Seq profiles of PU.1 in the same region. PU.1 is a hematopoietic Ets family member highly expressed in macrophages and showing a widespread association with enhancers (Ghisletti et al., 2010 [54]).
(0.98 MB TIF)

Figure S5 **Canonical lncRNA genes have a typical promoter chromatin signature at their 5′ end.** ChIP-Seq profiles at two representative genes, Malat1 (top) and Neat1 (bottom). The green box indicates a CpG island.
(0.80 MB TIF)

Figure S6 **Examples of extragenic Pol_II peaks in predicted enhancers overlapping annotated lincRNAs.** Four representative regions are shown. The H3K4me3/H3K36me3 domains from Guttman et al. [25] are indicated by red boxes, while enhancer predictions are indicated as black boxes.
(1.24 MB TIF)

Figure S7 **Correlation between total Pol_II and phospho-Ser5 Pol_II at extragenic regions with a promoter prediction.** The graphs display the distance between extragenic Pol_II peaks predicted as promoters/TSSs and the closest phospho-Ser5 Pol_II peak.
(0.27 MB TIF)

Figure S8 **Chromosome conformation capture (3C) assay at the Cxcl11 and Ccl5 loci.** The position of the anchor (constant) primer (red asterisk) and the Hind III restriction sites used is indicated. Inverted images of ethidium bromide-stained agarose gels are shown. n.s., non-specific band.
(0.55 MB TIF)

Table S1 **A curated dataset of extragenic Pol_II peaks in mouse macrophages.** Peaks were divided in constitutive, inducible, and repressed according to Pol_II behavior in response to stimulation.
(0.42 MB XLS)

Table S2 **Intergenic lncRNAs datasets used in this study.** The dataset termed “Ponjavic” is based on two datasets generated by the FANTOM consortium [26] and then filtered to eliminate all RNAs overlapping protein coding genes [24]. This led to a set of 3,122 macroRNAs that was further filtered against the current Ensemble protein coding gene annotations (mm_9) leading to 2,168 independent long ncRNAs. The dataset termed “Guttman” contains long non-coding RNA predicted on the base of H3K4me3/H3K36me3 chromatin signatures [25]. The original set is made up of 1,673 domains that were remap to mm9 and filtered against the current Ensemble protein coding gene annotations, leading to a final set of 1,408 long ncRNAs.
(0.39 MB XLS)

Table S3 **RNA Pol_II clusters overlapping annotated intergenic lncRNAs.** Clusters from Table S4 were overlapped with both datasets of lncRNAs in Table S2. The clusters and their matched lncRNAs are shown.
(0.13 MB XLS)

Table S4 **Promoter and enhancer predictions.** The extragenic Pol_II peaks were analyzed for chromatin signatures of enhancers or promoters using a machine-learning algorithm (described in the methods section). The table shows the prediction for each Pol_II peak. Peaks whose prediction was precluded are grouped. The table also shows the final dataset used for most of the analysis that resulted from a clustering and filtering procedure (described in the Methods section) of the Pol_II peaks predicted as promoters or enhancers. For each cluster the annotation of the closest neighboring UCSC known gene as well as the total number of RNA-seq transcripts, Q-PCR amplicons, and RNA repeats are shown.
(1.29 MB XLS)

Table S5 **Association between the transcriptional activity of enhancer-type extragenic Pol_II clusters with the expression of the associated protein-coding genes.** Extragenic Pol_II peak clusters with a signature of enhancer were assigned to the neighboring protein coding gene when distant less than 20 kb. Transcriptional activity of the assigned coding gene was evaluated on the basis of the Pol_II tag counts at ±500 bp surrounding their TSS in untreated and LPS-treated macrophages.
(0.09 MB XLS)

Table S6 **Phospho-Ser5 Pol_II datasets.** Peaks detected in untreated as well as LPS treated (2 h) macrophages against their input are listed.
(9.40 MB XLS)

Table S7 **Validation of 100 ncRNAs associated with predicted enhancers.** The table shows the genomic location of the regions, the Q-RT-PCR data, the sequence of the primers used, and the corresponding peak in Table S4.
(0.03 MB XLS)

Table S8 **Enrichment of TFBSs in the datasets of predicted enhancers and promoters.** The table shows the results of the Clover analysis carried out using a library of 338 high-quality PWMs (130 from the Jaspar database and 208 from Badis et al. 2009 [68]). The Summary shows the TFBS (PWMs) that are over-represented in each of the six individual groups (constitutive, inducible, and repressed enhancers; constitutive, inducible, and repressed promoters). In the other sheets the complete data for each group are shown. Each row represents a Pol_II peak (after a filtering step to eliminate nearby opposite predictions, see Methods section), while columns represent the enriched motifs. The Clover scores for every matrix and every peak are indicated. Matrices from the Bulyk group [68] are indicated by the prefix BU, while Jaspar matrices are preceded by the prefix MA.
(2.38 MB XLS)

Table S9 **Primers used in this study.** Primers used are shown with reference to each figure.
(0.04 MB XLS)

Table S10 **Regions used in the luciferase assay and relative cloning primers.**
(0.02 MB XLS)

Text S1 **Computational methods.** Chromosome conformation capture (3C) assay.
(0.08 MB DOC)

## Abbreviations

CAGE: cap analysis gene expression
ChIP: chromatin immunoprecipitation
CTD: C-terminal domain
H3K4me1: monomethylation of histone H3 at lysine 4
H3K4me3: trimethylation of histone H3 at lysine 4
IEG: immediate early gene
IL-6: interleukin 6
IRFs: interferon regulatory factors
lincRNA: large intervening non-coding RNA
LCR: locus control region
lncRNA: long non-coding RNA
ncRNA: non-coding RNA
Pol_II: RNA polymerase II
PRE: polycomb group response element
PWM: position weight matrix
TF: transcription factor
TFBS: transcription factor binding site
TSS: transcription start site
