# Supplementary figures and images for: A Large Fraction of Extragenic RNA Pol II Transcription Sites Overlap Enhancers
Source: PLoS Biol. 2010 May 11;8(5):e1000384. doi: 10.1371/journal.pbio.1000384 (PMC2867938; doi:10.1371/journal.pbio.1000384)

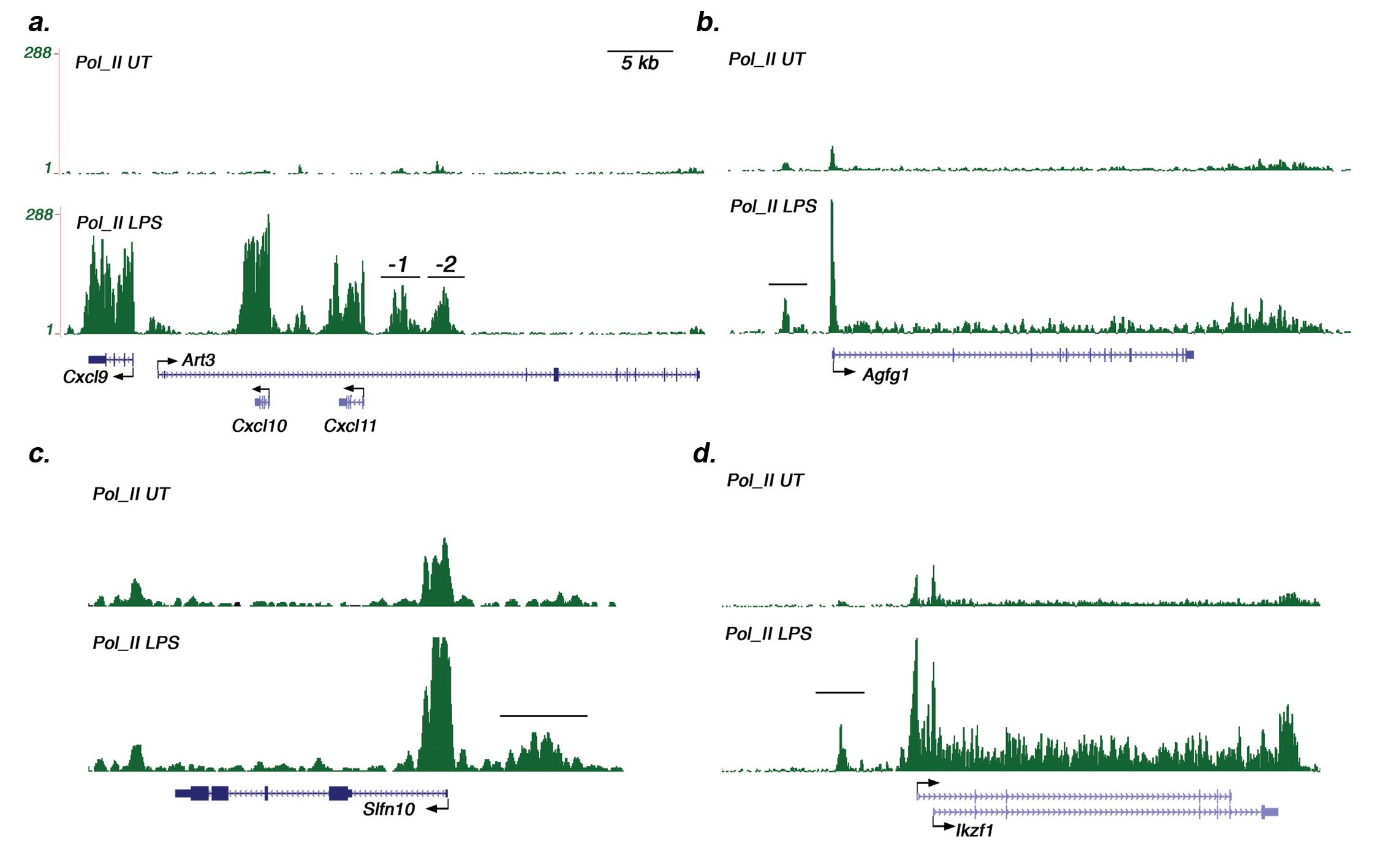

Supplement: Figure S1 — Inducible extragenic Pol_II peaks occurring upstream of LPS-inducible genes. (A) The Cxcl9-Cxcl11 chemokine gene clusters with two Pol_II peaks upstream of Cxcl11 highlighted. (B–D) Three additional representative genomic regions are shown. The extragenic Pol_II peaks are indicated by horizontal lines. Peaks can also be detected at lower levels in unstimulated macrophages. (0.55 MB TIF) [file pbio.1000384.s001.tif]

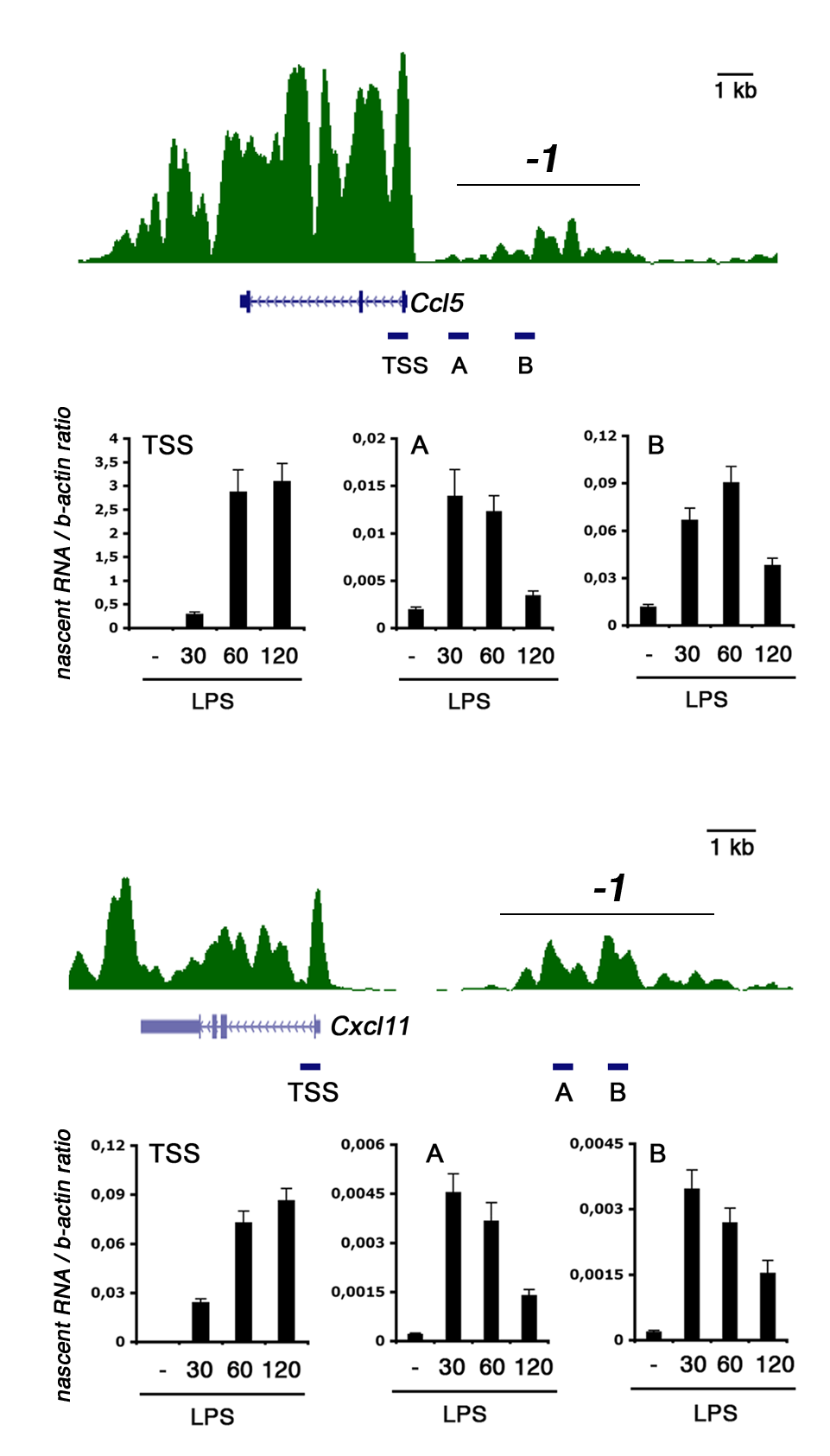

Supplement: Figure S2 — Nascent, chromatin associated transcripts at the Ccl5 (top) and Cxcl11 (bottom) loci were measured in LPS+γIFN-stimulated cells as indicated. The amplicons indicated are: TSS (transcription start site); A and B (corresponding to two regions contained within the −1 peak in Figure 1 [for Ccl5] and the −1 peaks in Figure S1A [for Cxcl11]). Pol_II ChIP-Seq data in the same regions (2h LPS+γIFN stimulation) are also shown. Error bars, s.e.m. (0.25 MB TIF) [file pbio.1000384.s002.tif]

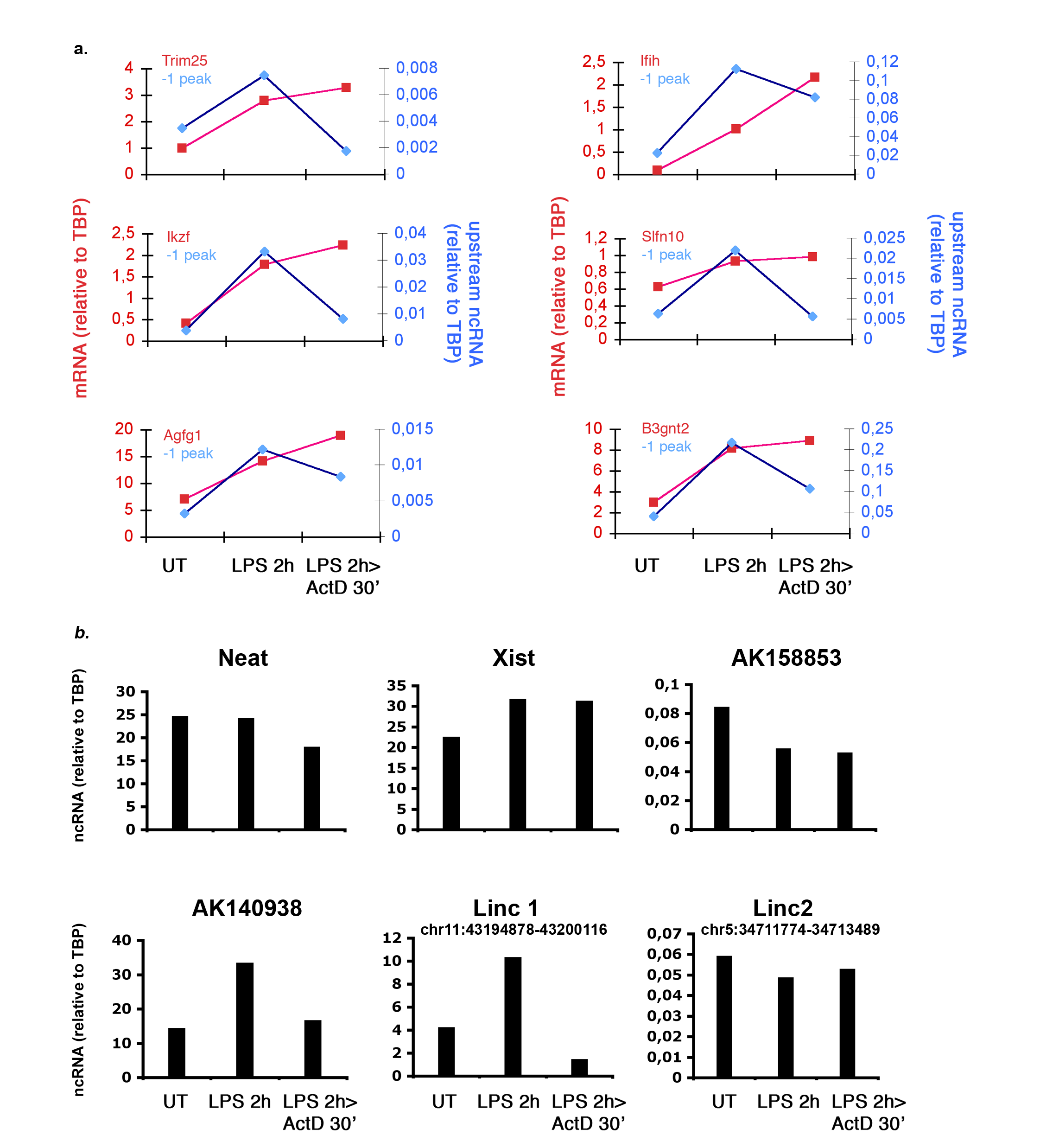

Supplement: Figure S3 — Stability of representative RNAs originating from extragenic Pol_II transcription sites. (A) Macrophages were stimulated with LPS for 2 h and then treated for 30 min with actinomycinD (ActD). Stability of the upstream non-coding transcripts is compared to that of the neighboring protein-coding gene. At each panel the y-axis on the left (in red) indicates the mRNA levels relative to those of the housekeeping gene TBP, while the y-axis on the right (light blue) indicates the levels of the neighboring upstream RNA generated by extragenic transcription. (B) Stability of annotated ncRNAs, including Neat, Xist, two Fantom transcripts, and two Linc RNAs. (0.61 MB TIF) [file pbio.1000384.s003.tif]

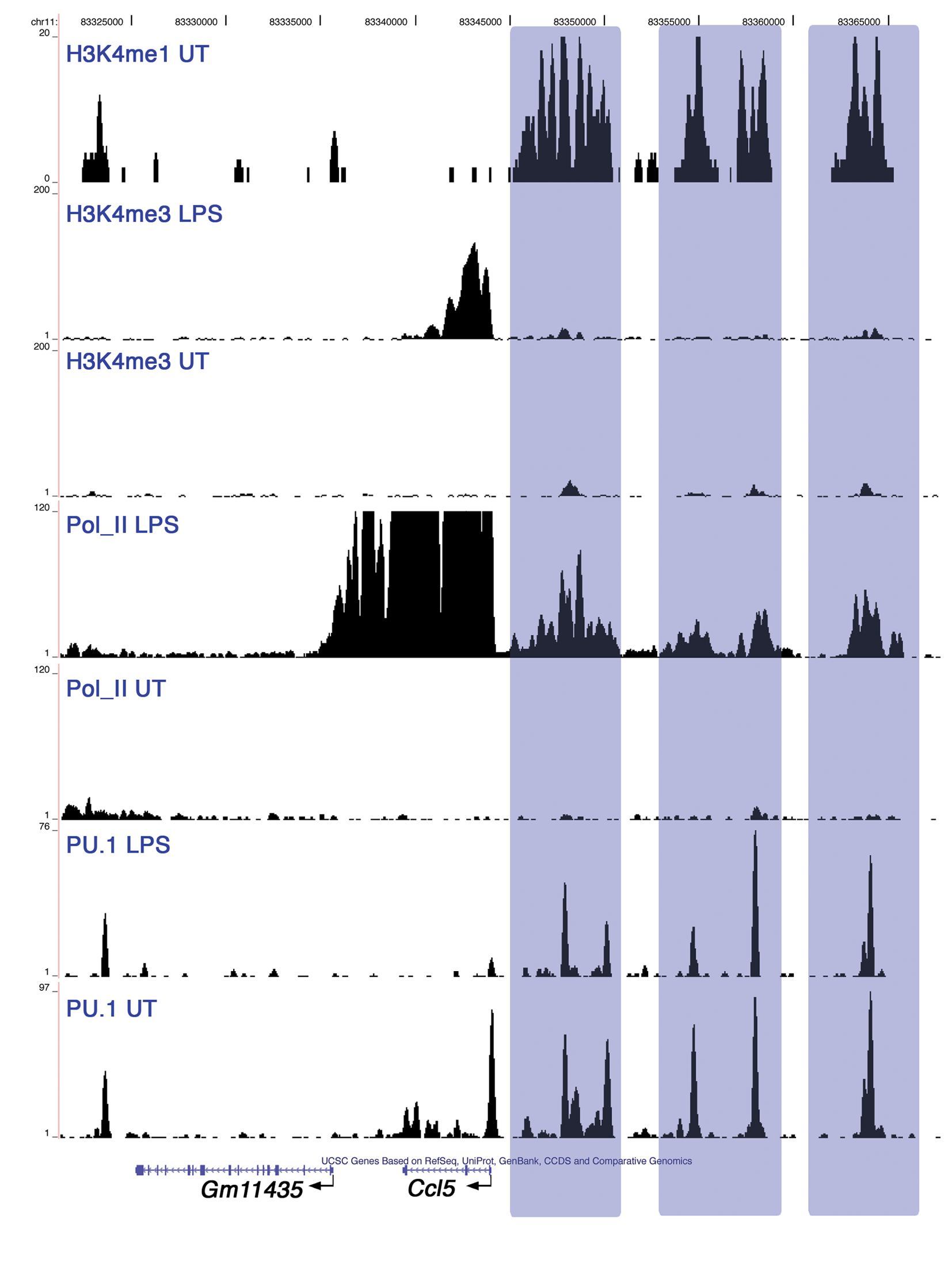

Supplement: Figure S4 — An enhancer-associated chromatin signature in the transcribed region upstream of Ccl5. The three main sites of extragenic transcription are indicated by shaded blue boxes. The two tracks at the bottom show the ChIP-Seq profiles of PU.1 in the same region. PU.1 is a hematopoietic Ets family member highly expressed in macrophages and showing a widespread association with enhancers (Ghisletti et al., 2010 [54]). (0.98 MB TIF) [file pbio.1000384.s004.tif]

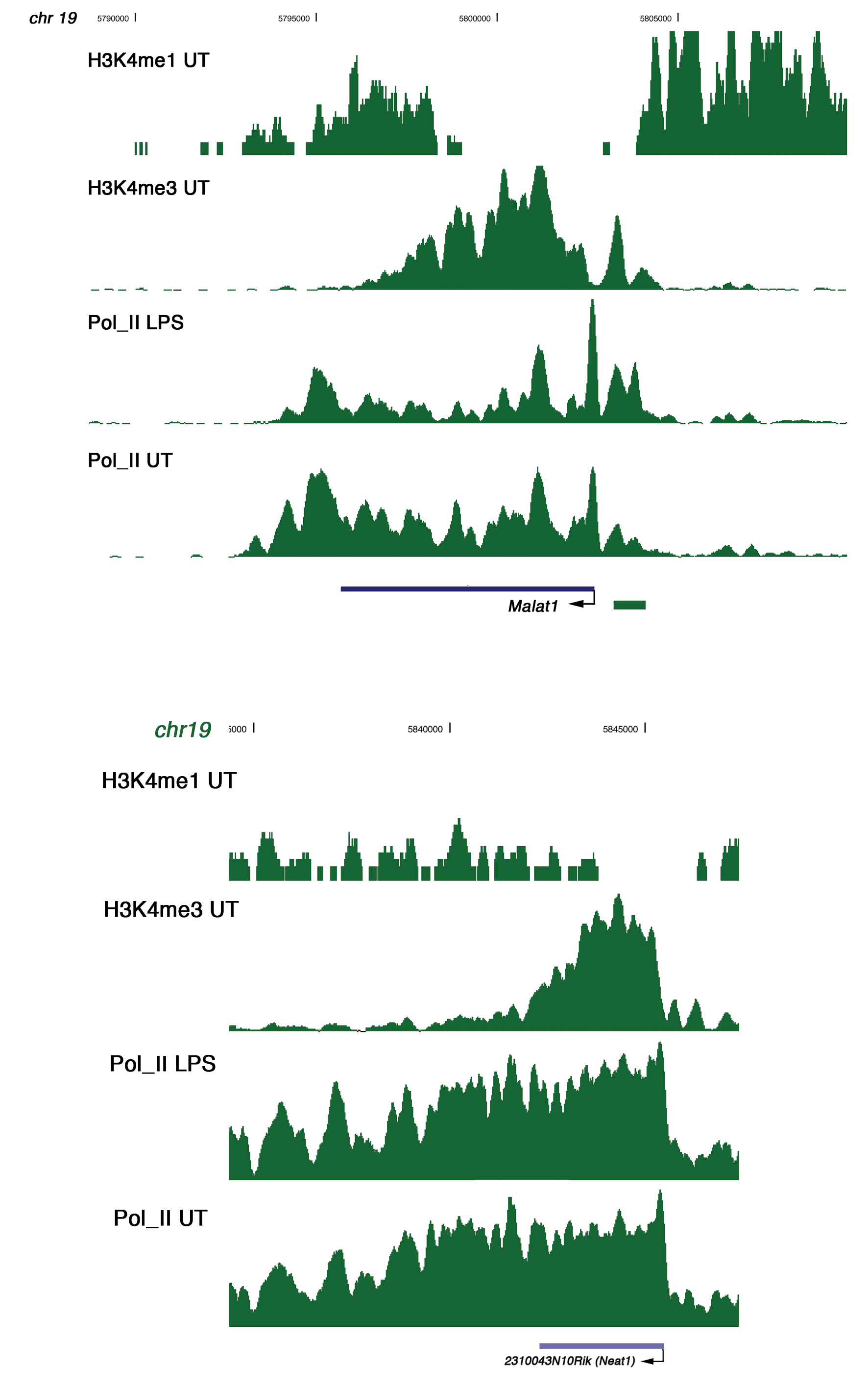

Supplement: Figure S5 — Canonical lncRNA genes have a typical promoter chromatin signature at their 5′ end. ChIP-Seq profiles at two representative genes, Malat1 (top) and Neat1 (bottom). The green box indicates a CpG island. (0.80 MB TIF) [file pbio.1000384.s005.tif]

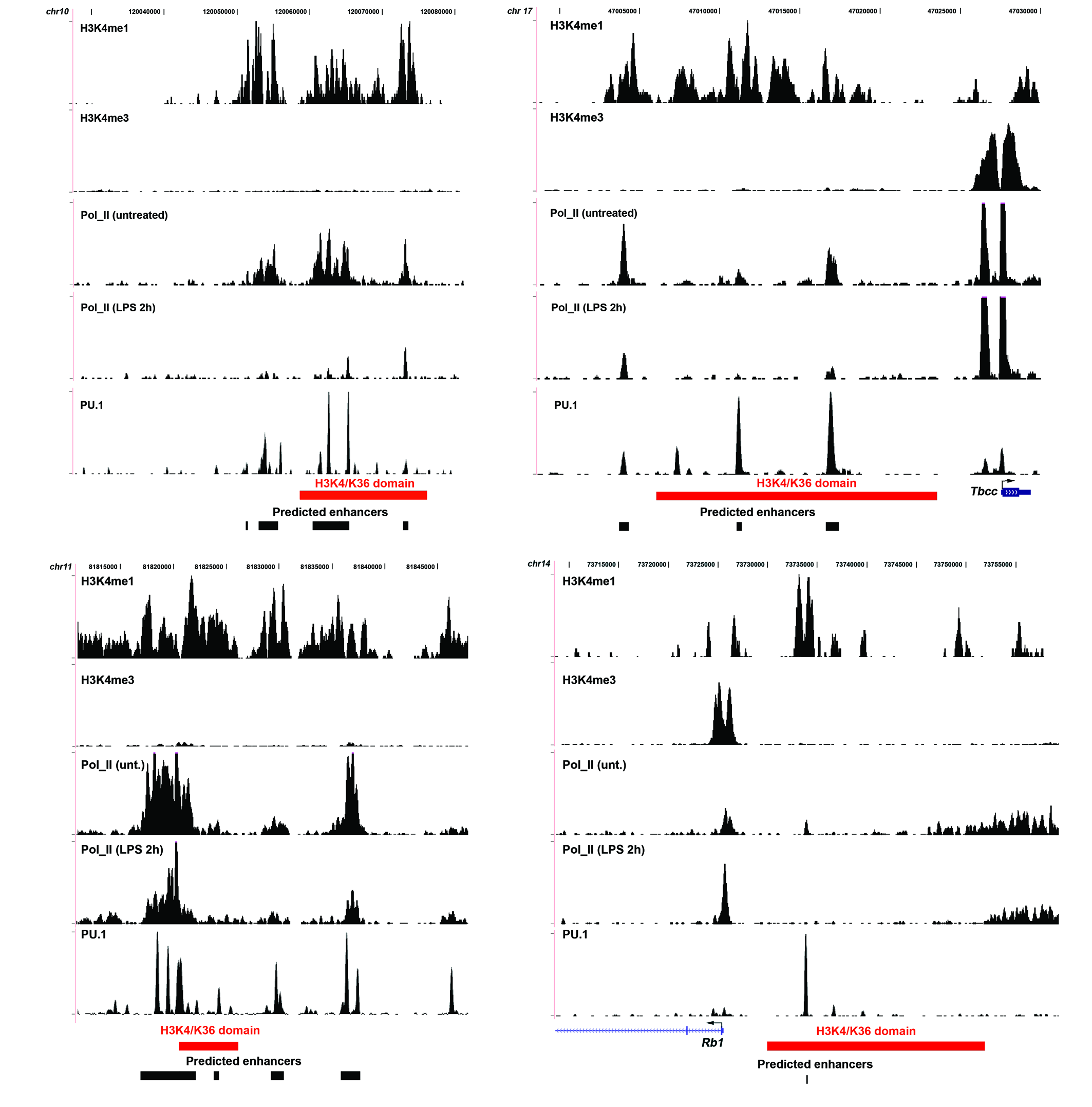

Supplement: Figure S6 — Examples of extragenic Pol_II peaks in predicted enhancers overlapping annotated lincRNAs. Four representative regions are shown. The H3K4me3/H3K36me3 domains from Guttman et al. [25] are indicated by red boxes, while enhancer predictions are indicated as black boxes. (1.24 MB TIF) [file pbio.1000384.s006.tif]

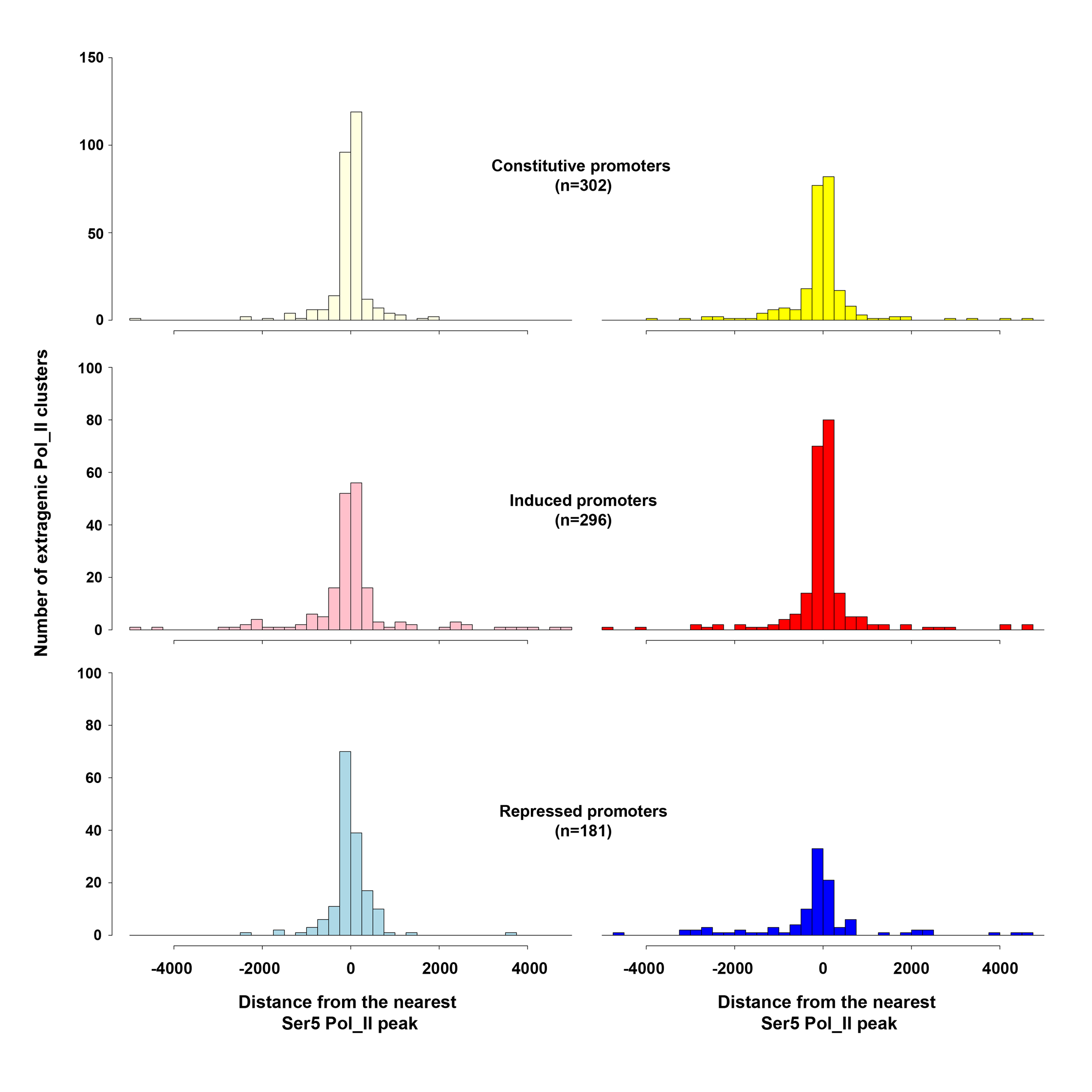

Supplement: Figure S7 — Correlation between total Pol_II and phospho-Ser5 Pol_II at extragenic regions with a promoter prediction. The graphs display the distance between extragenic Pol_II peaks predicted as promoters/TSSs and the closest phospho-Ser5 Pol_II peak. (0.27 MB TIF) [file pbio.1000384.s007.tif]

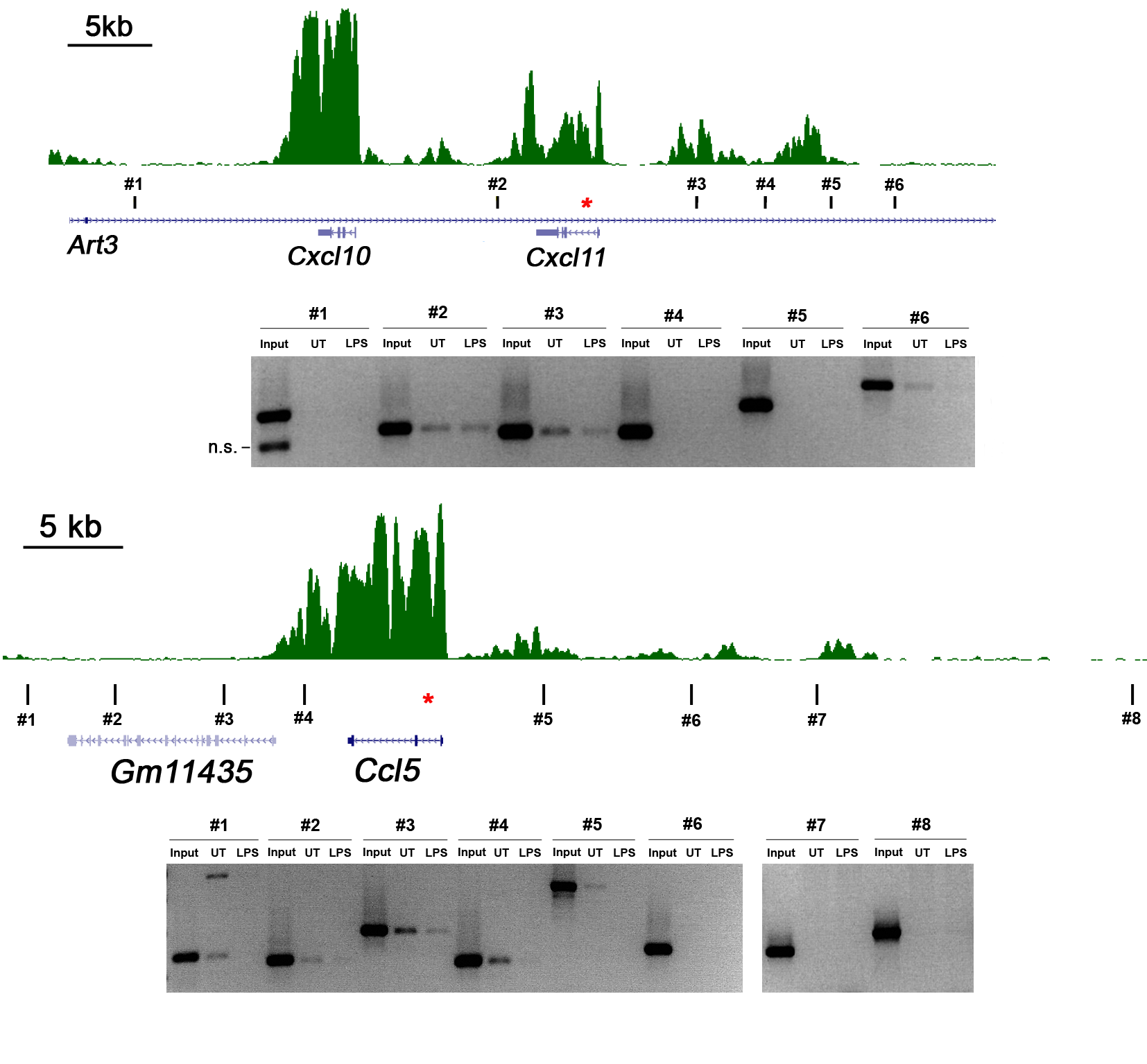

Supplement: Figure S8 — Chromosome conformation capture (3C) assay at the Cxcl11 and Ccl5 loci. The position of the anchor (constant) primer (red asterisk) and the Hind III restriction sites used is indicated. Inverted images of ethidium bromide-stained agarose gels are shown. n.s., non-specific band. (0.55 MB TIF) [file pbio.1000384.s008.tif]
