# Supplementary material for: A Large Fraction of Extragenic RNA Pol II Transcription Sites Overlap Enhancers
Source: PLoS Biol. 2010 May 11;8(5):e1000384. doi: 10.1371/journal.pbio.1000384 (PMC2867938; doi:10.1371/journal.pbio.1000384)
Supplement: Text S1 — Computational methods. Chromosome conformation capture (3C) assay. (0.08 MB DOC) [file pbio.1000384.s019.doc]

**Supplementary methods**

**Computational methods**

**Analysis of ChIP-Seq data**. The H3K4me3 and Pol_II datasets [5] and the H3K4me1 dataset (described in Ghisletti et al, in press) were aligned to the mm_9 mouse genome release using Bowtie [6]. In order to get unique matching tags (2 mismatches max.), we used the following parameters when invoking Bowtie from the command line: -a -v 2 -m 1 --best. The datasets were analyzed using MACS [7]. We obtained a list of regions constitutively marked by H3K4me3 or H3K4me1 through a statistical analysis of the corresponding dataset in untreated macrophages as compared to the input DNA from the same cells. The same approach was carried out for the Pol_II datasets. MACS was also run to compare the basal Pol_II dataset with the one obtained in stimulated macrophages. In all cases, MACS was run using a *p*-value threshold of 1e-5, and bw option set to 150. The phospho Ser5 Pol_II reads were generated at 76-bp. We used Bowtie [6] in order to align just the uniquely mappable reads (2 mismatches max.) onto the mm_9 genome. Because of the low quality of the bases at the 3' end of the reads, we trimmed them at different lengths in order to obtain the highest number of mappable reads onto the genome. This way, only the first 30bp at the 5' end for the untreated sample and the first 45bp at the 5' end for the LPS treated sample were kept. MACS was run to obtain a list of peaks in both samples normalizing against input DNA (*p*-value 1e-5).

**Using Support Vector Machines (SVMs) to discriminate between promoter-associated and enhancer-associated chromatin signatures.** Our aim was to find a machine-learning approach able to distinguish between two different classes of genomic elements (enhancers and promoters) based on their H3K4me1 and H3K4me3 profiles. We chose to use SVM perf, an optimized linear classifier based on a Support Vector Machine [8]. Given two distinct datasets represented by n-dimensional vectors, an SVM is able to construct a separating hyperplane, which maximizes the margin between the two datasets. This hyperplane may then be used to classify unknown data described by vectors of the same type.

In order to create a training set as unbiased as possible, we selected 556 regions (representative of the enhancers class) corresponding to +/- 2.5kbp surrounding the summit of validated extragenic p300 peaks (Ghisletti et al. in press) and bearing an informative signature; the promoter group was represented by an identical number of regions corresponding to +/- 2.5kbp surrounding the summit of RNA Pol_II peaks associated with mapped TSSs.

In more detail, in the case of the enhancer set we chose only regions showing at least an overlap with a statistically significant peak of H3K4me1 or H3K4me3. For the promoter group, we chose H3K4me3-positive regions overlapping a RefSeq TSS (in mm9 mouse genome alignment). This way, we selected informative regions for both classes.

H3K4me1 and H3K4me3 peaks were identified (using an input DNA from bone marrow macrophages as a reference) using MACS [7]. The *p*-value threshold was set to 1e-5 (--nomodel option on and bw option set to 150).

Furthermore, among all the informative regions we selected a subset of them displaying heterogeneous levels of H3K4me1 and H3K4me3. This way we obtained a training set with the maximal information content possible on the basis of the available data.

The 5kb regions identified through the procedure described above were split in 10bp windows and for each of them the number of overlapping tags from ChIP-seq experiments was computed. Because H3K4me1 and H3K4me3 datasets were sequenced at different levels of depth, the number of tags was linearly scaled in order to make them comparable.

This way, for each one of the 1'122 regions, we got two vectors of 500 elements, one for H3K4me1 and one for H3K4me3, that we combined together in a single vector. These vectors represented the input for the SVM.

We performed the training procedure using a different range of parameters for the SVM perf. To select the best combination of parameters, we evaluate the performance of the SVM using a leave-one-out cross-validation approach. That means that, given a certain training set (in our case 1'112 regions composed by 556 enhancers and 556 promoters), we ran 1'112 runs of cross-validation in which, in turn, 1'111 regions were used to train the SVM and 1 was tested.

We eventually used the default parameters except for --b set to 0, -l set to 2 and -c (the tradeoff parameter) set to 1. This way, the method reached an accuracy ((TP+TN)/(TP+FP+TN+FN)) of 0.97 with a specificity (TP/(TP+FN)) of 0.973 and a sensitivity (TN/(TN+FP)) of 0.968. We considered as true positives (TP) all enhancers that were correctly predicted; false positives (FP) the true enhancers that were incorrectly identified as promoters; true negatives (TN) the correctly predicted promoters; and false negatives (FN) the promoters that were incorrectly classified as enhancers. Once the best parameters were identified, the SVM was trained with all the 1'122 regions. The model obtained this way was then used to distinguish between promoter-like and enhancer-like regions among the 4'588 extragenic Pol_II peaks in our dataset. We initially centered the peaks on their summit and fetched the surrounding 5kb (+/- 2.5kb). We then retrieved the H3K4me1 and H3K4me3 levels - as described above. We finally filtered out the uninformative regions, defined as regions that neither overlaps a significant H3K4me1 or H3K4me3 peak. This way we identified 357 regions whose chromatin signature didn’t allow a prediction. H3K4me1/me3 tag counts were retrieved using custom C++ scripts. SVM perf cross-validation procedure, as well as ad hoc scripts used to tune parameters, were written in Python.

**Chromatin-signature of Pol_II peaks.** The chromatin information fetched for the predictions was ordered from chromosome 1 to chromosome X and was then visualized as a heatmap. Before plotting, a pseudocount of 1 was added to each data points that were then log2-transformed. R was used to perform these operations.

**Filtering and clustering of the predicted Pol_II peaks.** Initially, for each pair of Pol_II peaks within 500 bp from each other that presented opposite predictions but the same Pol_II behaviour (constitutive, inducible, repressed,) we purged the Pol_II peaks with the weakest prediction (see SVM score, **Supp. Table 4**).

Through this procedure, the 3,227 Pol_II peaks with an enhancer prediction were purged to 3,171 (427 constitutive, 848 induced and 1896 repressed) while the 1,004 promoters to 832 (358 constitutive, 359 induced, 215 repressed).

The resulting regions that were within 1kbp from each other were clustered together. As a final step, we filtered the Pol_II clusters against the Ensembl protein-coding dataset (mm_9, downloaded on January 20^th^, 2009). We considered as protein-coding all the regions annotated as “protein_coding”, “IG_C_gene” or “IG_V_gene” and used this information for filtering (final clusters are listed in **Supp. Table 4**).

The same procedure (except the first purging step) was applied to the dataset of unpredictable peaks.

For all analyses we used the final clusters, with two exceptions. For transcriptional and conservation analyses, in which information at single base-pair resolution was needed, we used the unclustered Pol_II peaks. In order to avoid any bias coming from the potential protein-coding sequences, we filtered the peaks against the Ensembl protein-coding dataset. This procedure resulted in 3105 enhancers-predicted peaks, 885 promoters-predicted and 328 unpredictable regions.

**Analysis of enrichment of CpG islands in the datasets.** Coordinates of annotated CpG islands were retrieved from the mm9 mouse genome release (downloaded from UCSC on July 20^th^, 2009). We first computed the overlaps between these regions and our clusters of predicted enhancers and promoters. The overlaps we found were then statistically evaluated. For each dataset, we generated (using Python scripts) 1'000 random sets of extragenic regions with the same distribution of lengths. We then overlapped these random sets against the annotated CpG islands and used them to build a distribution. We finally computed a *p*-value for the observed overlaps, defined as the number of random overlaps exceeding the observed overlap on the total number of random sets evaluated.

**External ncRNAs datasets, re-mapping and filtering.** The first dataset we used is based on two datasets generated by the FANTOM consortium [2] and then filtered to eliminate all RNAs overlapping protein coding genes [3]. This led to a set of 3,122 macroRNAs that was re-mapped to mm_9 and further filtered against the current Ensemble protein-coding gene annotations leading to 2,168 independent long ncRNAs. The second dataset contains long non-coding RNA predicted on the base of H3K4me3/H3K36me3 chromatin signatures [1]. The original set consisted of 1'673 domains that were re-mapped to mm_9 and filtered against the current Ensemble protein-coding gene annotations, leading to a final set of 1'408 long ncRNAs.

**Analysis of sequence conservation.** phastCons scores [9] computed among 20 placental mammals were fetched from the mm9 mouse genome release. We retrieved scores at a single base pair resolution for all the 5kb regions surrounding the summit of extragenic Pol_II peaks identified in this study after the initial step of purging but before clustering. We considered each class (promoter-type, enhancer-type and uninformative regions) separately.

We also generated a dataset of 4,318 random extragenic regions of identical length (5kb). We then retrieved the corresponding phastCons scores at base pair resolution. For each class and for the random set, we then computed the mean of the phastCons scores for each position of the 5kb window and plotted the result obtained. At the same time, a t-test was performed for each base pair in the 5kb range, comparing the distributions of phastCons scores in each class with those of the random set. PhastCons scores were retrieved using custom C++ scripts. Statistical analyses and plots were generated with R.

**Analysis of TFBS enrichment in datasets.** TFBS over-representation in each dataset (inducible, repressed and constitutive enhancers or promoters) was evaluated using CLOVER [10]. Given a set of PWMs describing known transcription factor DNA binding sites, and coherent background sets of sequences, CLOVER assesses which PWMs are significantly over- and under-represented in a set of input sequences. CLOVER was run with a *p*-value threshold of 0.01 and with the verbose option on (with the other parameters set to default). Using the verbose option, CLOVER prints, besides the usual output (a list of over- and under- represented motifs), the single raw score contributions for each input sequences. For each experimental sequence and a statistically significant PWM identified in it, a score representing the likelihood that the sequence contains one or more binding sites corresponding to the PWM is returned. We used these scores to build a matrix and a heatmap. Two background sets were obtained from the mm9 mouse genome: the first one consisted of the entire chromosome 19, while the second one was composed of the 5 kb upstream of the TSSs of all the annotated RefSeq. We considered only the PWMs that CLOVER reported significant (with a *p*-value lower than 0.01) for both the background sets. As PWMs dataset, we used 130 PWMs from the last release of the JASPAR database [11] and 208 experimentally determined PWMs from Bulyk and coworkers (2 PWMs for each of the 104 TF tested) [4]. The final heatmap was drawn using R starting from the data parsed from the CLOVER output.

**CAGE tags analyses.** Mouse CAGE tags clusters details (PMID: 19374775) were downloaded from the Fantom4 website (<http://fantom.gsc.riken.jp/4/download/Tables/mouse/CAGE/promoters/tag_clusters/>). Clusters with coming from all the available tissues were pooled together resulting in 2'651'762 clusters.

Considering each one of our regions, the median of the distances between the CAGE tags clusters overlapping it represents a measure of the spread of the CAGE tags clusters onto it. This information was calculated for each region using C++ scripts and then the distribution of the medians was box-plotted separately for enhancers-like and promoters-like clusters.

**RNA sequencing.** Sequence reads coming from four independent Illumina Genome Analyzer II lanes were pooled together and mapped to the mouse mm9 genome using TopHat [12]. All reads that mapped uniquely to the genome (-g 1 options on the TopHat command line) with two or fewer mismatches were kept. Two out of four lanes were sequenced up to 76bp while the remaining two up to 55bp. For this reason we trimmed all the reads to 55bp. TopHat is able to find de novo splice junction and to use a table of annotated splice junctions at the same time. We provided TopHat with a table of known splice junctions derived from Ensembl

(<ftp://ftp.ensembl.org/pub/current_gtf/mus_musculus/>).

Transcripts discovery and quantification was performed using Cufflinks 0.8.0 (<http://cufflinks.cbcb.umd.edu/>). The RNA repeats track for the mm9 mouse genome was donwloaded from the UCSC genome browser (on January 20^th^, 2009) and used to filter out rRNA, scRNA, snRNA, srpRNA and tRNA overlapping out extragenic Pol_II sites.

**Chromosome conformation capture (3C) assay.**

Bone marrow-derived primary macrophages (5x10^6^), untreated or stimulated with LPS and γIFN, were fixed in 3 ml of medium with 1.5% formaldehyde for 10 min at room temperature. The reaction was quenched by the addition of Tris-HCl pH 7.6 to a final concentration of 125mM and cells were then washed with three times PBS 1X. Cells were pelleted and resuspended in 6 ml of 4°C cold cell lysis buffer (10mM Tris pH 8.0, 10 mM NaCl, 0.2% NP-40 and protease inhibitors) and incubated on ice for 30 min (with shaking using a small magnetic stirrer). Nuclei were then centrifuged at 1500 rpm and resuspended in 250 μl of 1.2X standard Hind III buffer (60 mM NaCl, 12 mM Tris pH 7.9, 1.2 mM DTT). 20% SDS was added to a final concentration of 0.3% and nuclei were incubated in a thermo-mixer at 37°C for 1h to extract non-crosslinked proteins from the DNA. 20% Triton X-100 was then added to a final concentration of 1.8% and the nuclei were incubated at 37°C for 1h to sequester SDS and allow subsequent digestion. Aliquots of nuclei corresponding to 10^6^ cells were digested with 200U of concentrated Hind III (New England Biolabs); the digestion was performed at 37°C overnight. The enzyme was inactivated by the addition of 20%SDS to a final concentration of 1.3% and incubation at 65°C for 20 min. One reaction was saved in order to check the digestion efficiency by QPCR using non-crosslinked non-digested genomic DNA as reference; each of the other reactions was splitted in 7 aliquots and each aliquot was made up to 800 μl with ligation buffer (30 mM Tris pH 8.0, 10 mM MgCl_2_, 10 mM DTT, 1mM ATP) containing 1% Triton X-100. 500U of NEB T4 DNA ligase were added and the ligation was incubated at 16°C for 5h and then at room temperature for additional 30 min. The samples, both ligated and not ligated, were treated with Proteinase K to a final concentration of 100 μg/ml and incubated at 65°C overnight to reverse the formaldehyde crosslinks. The following day, the DNA was purified by phenol extraction and ethanol precipitation using glycogen as carrier. The aliquots were pooled and the samples dissolved in 400 μl (total) of TE 1X. 5μl of this preparation were used for PCR amplification (35 cycles) and PCR products were run on a 2% agarose gel.

The reference library was generated by PCR amplification of non-crosslinked genomic DNA using primers designed around the Hind III sites of interest. The PCR fragments were then purified and quantified. 30 ng of each fragment were digested with Hind III at 37°C for 2h; the reaction was inactivated by incubation at 70°C for 10 min. The ligation was performed by incubating 1/10 of each Hind III-digested PCR product and 2 μg of Hind III-digested genomic DNA with 2000U of T4 DNA ligase in 100 μl total at 16°C overnight. 2μl of this ligation were used for PCR amplification using the primers indicated in **Suppl. Table 7** and PCR products (Input) were run on a 2% agarose gel.

**Supplementary references**

1. Guttman M, Amit I, Garber M, French C, Lin MF, et al. (2009) Chromatin signature reveals over a thousand highly conserved large non-coding RNAs in mammals. Nature 458: 223-227.

2. Carninci P, Kasukawa T, Katayama S, Gough J, Frith MC, et al. (2005) The transcriptional landscape of the mammalian genome. Science 309: 1559-1563.

3. Ponjavic J, Ponting CP, Lunter G (2007) Functionality or transcriptional noise? Evidence for selection within long noncoding RNAs. Genome Res 17: 556-565.

4. Badis G, Berger MF, Philippakis AA, Talukder S, Gehrke AR, et al. (2009) Diversity and Complexity in DNA Recognition by Transcription Factors. Science 324: 1720-1723.

5. De Santa F, Narang, V., Yap, ZH., Khoramian Tusi, B., Burgold, T., Austenaa, L., Bucci, L., Caganova, M., Notarbartolo, S., Casola, S., Testa, G., Sung, WK., Wei, CL., Natoli, G. (2009) Jmjd3 contributes to the control of gene expression in LPS-activated macrophages. EMBO J 28: 3341-3352.

6. Langmead B, Trapnell C, Pop M, Salzberg SL (2009) Ultrafast and memory-efficient alignment of short DNA sequences to the human genome. Genome Biol 10: R25.

7. Zhang Y, Liu T, Meyer CA, Eeckhoute J, Johnson DS, et al. (2008) Model-based analysis of ChIP-Seq (MACS). Genome Biol 9: R137.

8. Joachims T (2005) A Support Vector Method for Multivariate Performance Measures. Proceedings of the International Conference on Machine Learning (ICML).

9. Siepel A, Bejerano G, Pedersen JS, Hinrichs AS, Hou M, et al. (2005) Evolutionarily conserved elements in vertebrate, insect, worm, and yeast genomes. Genome Research 15: 1034-1050.

10. Frith MC, Fu Y, Yu L, Chen JF, Hansen U, et al. (2004) Detection of functional DNA motifs via statistical over-representation. Nucleic Acids Res 32: 1372-1381.

11. Sandelin A, Alkema W, Engstrom P, Wasserman WW, Lenhard B (2004) JASPAR: an open-access database for eukaryotic transcription factor binding profiles. Nucleic Acids Res 32: D91-94.

12. Trapnell C, Pachter L, Salzberg SL (2009) TopHat: discovering splice junctions with RNA-Seq. Bioinformatics 25: 1105-1111.
